# Non-Nucleotide RNA-Dependent RNA Polymerase Inhibitor That Blocks SARS-CoV-2 Replication

**DOI:** 10.3390/v13081585

**Published:** 2021-08-11

**Authors:** Milan Dejmek, Eva Konkoľová, Luděk Eyer, Petra Straková, Pavel Svoboda, Michal Šála, Kateřina Krejčová, Daniel Růžek, Evzen Boura, Radim Nencka

**Affiliations:** 1 Institute of Organic Chemistry and Biochemistry, Czech Academy of Sciences, Flemingovo náměstí 542/2, 160 00 Praha, Czech Republic; dejmek@uochb.cas.cz (M.D.); eva.konkolova@uochb.cas.cz (E.K.); sala@uochb.cas.cz (M.Š.); katerina.krejcova@uochb.cas.cz (K.K.); 2 Veterinary Research Institute, Emerging Viral Diseases, Hudcova 296/70, 621 00 Brno, Czech Republic; eyer@vri.cz (L.E.); strakova.p@centrum.cz (P.S.); svoboda@vri.cz (P.S.); 3 Institute of Parasitology, Biology Centre of the Czech Academy of Sciences, Branišovská 1160/31, 370 05 České Budějovice, Czech Republic; 4 Department of Pharmacology and Pharmacy, Faculty of Veterinary Medicine, University of Veterinary Sciences Brno, Palackého tř. 1946/1, 612 42 Brno, Czech Republic

**Keywords:** non-nucleotide inhibitor, RNA-dependent RNA polymerase, SAR-CoV-2, COVID-19, antiviral agents

## Abstract

SARS-CoV-2 has caused an extensive pandemic of COVID-19 all around the world. Key viral enzymes are suitable molecular targets for the development of new antivirals against SARS-CoV-2 which could represent potential treatments of the corresponding disease. With respect to its essential role in the replication of viral RNA, RNA-dependent RNA polymerase (RdRp) is one of the prime targets. HeE1-2Tyr and related derivatives were originally discovered as inhibitors of the RdRp of flaviviruses. Here, we present that these pyridobenzothiazole derivatives also significantly inhibit SARS-CoV-2 RdRp, as demonstrated using both polymerase- and cell-based antiviral assays.

## 1. Introduction

Coronaviruses are positive-sense RNA viruses that cause numerous important human and animal diseases. These viruses are classified into four genera including *Alphacoronavirus* and *Deltacoronavirus* [1]. While infectivity of gammacoronaviruses and deltacoronaviruses is limited to animals, mostly birds [2], alphacoronaviruses and betacoronaviruses comprise numerous mammalian and human pathogens [3,4]. Important alphacoronaviruses are Human coronaviruses (HCoVs) 229E and NL63, as well as Feline coronavirus (FIPV) and Porcine epidemic diarrhea virus (PEDV). Betacoronaviruses include the rest of human coronaviruses (HCoV-OC43, HCoV-HKU1, Severe acute respiratory syndrome coronavirus (SARS-CoV and SARS-CoV-2), Middle East respiratory syndrome coronavirus (MERS-CoV)) and a number of other animal pathogens. While coronaviruses that have persisted in the human population for a long time (HCoV-229E, HCoV-NL63, HCoV-OC43, and HCoV-HKU1) cause milder upper respiratory illnesses, SARS-CoV, SARS-CoV-2, and MERS-CoV are highly pathogenic viruses causing severe lower respiratory illness that can progress to life-threatening pneumonia with significant mortality rates [5,6].

SARS-CoV-2 is responsible for the largest pandemic of acute viral disease that our world has faced since the Spanish flu [7]. Although the mortality of this disease is not as high as in that of SARS-CoV or MERS-CoV infections, the disease caused by this virus (COVID-19) has significantly changed the world in which we live [8]. In particular, mortality in older age groups is alarming [9], and although we already have vaccines [10] as well as several drugs based on both monoclonal antibodies and small molecules, the effectiveness of these treatments is limited by several factors, including the high mutation rate of this virus [11]. Therefore, it is essential that pharmaceutical research focuses on the widest possible range of molecular targets and that we seek to find substances that we can use in combination with already known drugs to reduce the risk of the development of resistance. One of the main molecular targets in viruses is their polymerase, which is responsible for the replication of their genetic information. The example of HIV has shown that a combination of nucleoside and non-nucleoside reverse transcriptase inhibitors can lead to a very effective therapy for this serious disease [12]. Therapies for diseases caused by other viral pathogens, such as hepatitis B and C viruses (HBV and HCV), also rely largely on polymerase inhibitors [13,14]. Therefore, it is not surprising that the main approach for the development of new antivirals against SARS-CoV-2, as well as other coronaviruses, is based on targeting the RNA-dependent RNA polymerase (RdRp) as a central replication enzyme of the virus [15]. RdRp is part of the large coronaviral replication complex that also includes RNA methyltransferases, helicase, nsp9, and probably the N protein [16]. The coronaviral RdRp is a highly conserved heterotrimeric protein complex that is composed of two accessory but essential proteins (nsp7 and nsp8) and the catalytic subunit nsp12 [17]. While the first nucleoside-based RdRp inhibitor has already been approved by the relevant authorities around the world [18], information on potent non-nucleoside inhibitors of this key enzyme is rather scarce. Recently, suramin was reported as a potent non-nucleoside inhibitor of SARS-CoV-2 RdRp, and its mode of action was supported by a cryo-EM structure [19]. Also, several natural products including corilagin [20] and lycorine [21] exert inhibition potency against SARS-CoV-2 RdRp. Although efforts have been made to identify new non-nucleoside inhibitors of this enzyme by in silico methods since the beginning of the pandemic, the results of these studies are unfortunately rarely supported by experimental data [22].

HeE1-2Tyr (compound **16**) was initially identified by Tarantino et al. as a potent inhibitor of RdRp from Dengue virus (DENV), West Nile virus, and Yellow fever virus, all members of genus *Flavivirus* [23,24,25,26]. This compound was crystalized in complex with the RdRp from DENV 3. It was shown that the drug binds on the thumb side of the RNA-binding site, with significant movement of the priming loop. The authors also suggested that there is another possible binding site of the compound that is hidden by the priming loop. This hypothesis has been supported by point mutation experiments [23].

Here, we report on the identification of HeE1-2Tyr (**16**) and its derivatives as potential inhibitors of SARS-CoV-2 RdRp. The drugs exert activity not only against SARS-CoV-2 but also against FIPV. Our study started with the screening of our small collection of various nucleoside triphosphate and non-nucleoside inhibitors of polymerases from different viruses against SARS-CoV-2 RdRp and, in parallel, a screening of our complementary nucleoside, nucleotide prodrug, and non-nucleoside derivative library against SARS-CoV-2 in cell-based assays. To our surprise, we identified HeE1-2Tyr (**16**), which we prepared as a standard for our studies on flavivirus RdRps, as an effective inhibitor of SARS-CoV-2 RdRp that also exerted significant activity against the virus in cell cultures.

## 2. Material and Methods

### 2.1. Protein Expression and Purification

SARS-CoV-2 nsp7 (GeneBank: YP_009725303), nsp8 (GeneBank: YP_009725304,) and nsp12 (GeneBank: YP_009725307) genes were commercially synthesized as codon-optimized for *E. coli* (Invitrogen). The gene for nsp7 was cloned into a modified pRSF-Duet vector containing an N-terminal 6×His tag, followed by a GB1 solubility tag, a 10×Asp spacer sequence, and a tobacco etch virus (TEV) protease cleavage site in cloning site 1. The nsp8 gene was subsequently cloned into cloning site 2 without any tag. The gene for nsp12 was cloned into the pAceBac vector with cleavable 6×His on the C-terminus. The nsp7/nsp8 protein complex was expressed and purified as described previously for truncated nsp7/nsp8 in *E. coli* [27]. The SARS-CoV-2 nsp12 plasmid was used to prepare recombinant baculovirus Sf9 insect cells that were infected at 1–2 × 10^6^ cell/mL with the tertiary recombinant baculovirus. After 68 h, the cells were collected by centrifugation, resuspended in lysis buffer (50 mM HEPES 7.4, 300 mM NaCl, 20 mM imidazole, 3 mM MgCl_2_, 10% (*v/v*) glycerol, and 3 mM β-mercaptoethanol) and sonicated (Q700 Sonicator, QSonica). The lysate was subsequently cleared by centrifugation, and the supernatant was incubated with Ni-NTA agarose (Thermo Scientific) and washed with lysis buffer; finally, the protein was eluted with lysis buffer supplemented with 300 mM imidazole. Nsp12 protein was further purified by size-exclusion chromatography using Superdex 200 16/600 (GE Healthcare) in size-exclusion buffer (20 mM HEPES 7.4, 300 mM NaCl, 1 mM MgCl_2_, 10% (*v/v*) glycerol, and 3 mM β-mercaptoethanol). Fractions containing the pure nsp12 protein were concentrated to 5 mg/mL, flash-frozen, and stored at −80 °C until needed.

### 2.2. Fluorescence-Based Primer Extension Polymerase Assay

The polymerase activity was determined in a primer extension reaction using a fluorescently labeled primer (HEX-5′-AGAACCUGUUGAACAAAAGC-3′) and an RNA template (5′- AUUAUUAGCUGCUUUUGUUCAACAGGUUCU-3′). The polymerase activity assay was performed in a total volume of 10 μL containing the reaction buffer (10 mM Tris pH 8.0, 2 mM MgCl_2_, 10 mM KCl, 1 mM β-mercaptoethanol), 10 μM NTPs, 0.5 μM T/P complex, 1 μM nsp12 polymerase, and 3 μM nsp7/nsp8 complex. The reactions were incubated for 1 h with various concentrations of the inhibitors tested at 30 °C and stopped by adding 20 μL of the stop buffer (80% formamide, 50 mM EDTA). The samples were denatured at 95 °C for 10 min, and primer extension products were separated on a 20% denaturing polyacrylamide gel (8 M urea, 1× TBE, 20% acrylamide (19:1)) and scanned on a Typhoon 5 Biomolecular Imager (GE Healthcare).

### 2.3. Radioactivity-Based Primer Extension Polymerase Assay

The assay was performed as above, except that the reaction mixture contained 0.5 µM T/P complex (P-5′-AGAACCUGUUGAACAAAAGC-3′, T-5′-U25-GCUUUUGUUCAACAGGUUCU-3′) and 0.01µCi/µL [α-32P]-ATP. After incubation, 5 µL of the reaction mixtures was spotted on an anion-exchange cellulose filter paper (WhatmanTM Grade DE81 DEAE cellulose paper; GE Healthcare) in triplicates. The Whatman filter was then dried, subsequently washed with 0.125 mM Na_2_HPO_4_, water, and ethanol, and dried again. The dry filter paper was then analyzed using phosphorimaging, the plate was scanned on Amersham Typhoon 5 Biomolecular Imager (GE Healthcare), products were quantified with Image Studio Lite (LI-COR), and the data were processed using GraphPad version 6 (GraphPad Prism version 6, GraphPad Software, San Diego, CA, USA).

### 2.4. Viruses and Cell Lines

Two representatives of the *Coronaviridae* family, i.e., SARS-CoV-2 (strain SARS-CoV-2/human/Czech Republic/951/2020 isolated from a clinical sample at the National Institute of Health, Prague, Czech Republic, and kindly provided by Dr. Jan Weber, Institute of Organic Chemistry and Biochemistry, Prague, Czech Republic) and feline infectious peritonitis virus (FIPV, ATCC VR990, a pathogen of domestic cats and other felines), were used for our antiviral cell-based studies. The experiments with the live coronaviruses were performed in our BSL3 facility.

Vero cells (ATCC CCL-81, African Green Monkey, adult kidney, epithelial) and Vero E6 cells (ATCC CRL-1586) were cultured in Dulbecco′s modified Eagle′s medium (DMEM) supplemented with 10% newborn calf serum, 100 U/mL penicillin, 100 µg/mL streptomycin, and 1% glutamine (Sigma-Aldrich, Prague, Czech Republic) at 37 °C and 5% CO_2_. Colorectal adenocarcinoma cells (CaCo-2, ATCC HTB-37) were grown in DMEM medium, containing, 20% newborn calf serum with 100 U/mL penicillin, 100 µg/mL streptomycin, and 1% L-glutamine (Sigma-Aldrich, Prague, Czech Republic) at 37 °C and 5% CO_2_. *Felis catus* kidney cortex cells (CRFK, ATCC CCL-94) were grown in DMEM supplemented with 10% newborn calf serum, 100 U/mL penicillin, 100 µg/mL streptomycin, and 1% glutamine (Sigma-Aldrich, Prague, Czech Republic) at 37 °C and 5% CO_2_. Vero (ATCC CCL-81), CaCo-2 (ATCC HTB-37), and CRFK (ATCC CCL-94) cells were used for antiviral and cytotoxicity assays, and Vero E6 cells (ATCC CRL-1586) were used to perform plaque assays.

### 2.5. Cytotoxicity Studies

Vero (ATCC CCL-81), CaCo-2 (ATCC HTB-37), and CRFK (ATCC CCL-94) cells were seeded into each well of 96-well microtitrate plates (approx. 2 × 10^4^ cells per a well) and were incubated for 24 h at 37 °C and 5% CO_2_. Cell monolayers in 96-well plates were treated with the compounds remdesivir, HeE1-2Tyr (**16**), **17**, or **18** at the concentration of 50 μM or with 1% DMSO (*w*/*w*) (for the initial screening; Vero and CRFK cell lines) or with the compounds remdesivir, HeE1-2Tyr (**16**) and **17** in concentration ranges from 0 to 50 μM (for dose–response studies; 2-fold dilutions, three wells per concentration; Vero, CaCo-2, and CRFK cell lines) and cultured for 48 h. The cytotoxic activity of the compounds was determined in terms of cell viability using a Cell Counting Kit-8 (Dojindo Molecular Technologies, Munich, Germany) following the manufacturer’s instructions. The assay is based on the quantitative reduction of WST-8 tetrazolium salt to yellow formazan by cellular dehydrogenases. Cell viability was estimated as the percentage of colorimetric absorbance at 450 nm of the compound-treated cells relative to the absorbance of mock-treated cells. The concentration of compound that reduced cell viability by 50% was considered the 50% cytotoxic concentration (CC_50_).

### 2.6. Antiviral Efficacy of the Studied Compounds in Cell-Based Assays

To study the antiviral effects of compounds HeE1-2Tyr (**16**), **17**, and **18**, we used a viral titer reduction assay. Vero (ATCC CCL-81), CaCo-2 (ATCC HTB-37), and CRFK (ATCC CCL-94) cells were seeded into each well of 96-well microtitrate plates (approx. 2 × 10^4^ cells per a well) and incubated for 24 h at 37 °C and 5% CO_2_. Then, the medium was aspirated, replaced with 200 µL of fresh medium containing compounds HeE1-2Tyr (**16**), **17**, or **18** at the concentration of 50 μM (for the initial screening) or with compounds HeE1-2Tyr (**16**) or **17** in the concentration range of 0 to 50 µM (for dose–response antiviral studies; 2-fold dilution, three wells per compound). The treated cells were simultaneously inoculated with SARS-CoV-2 (for Vero and CaCo-2 cells) or FIPV (for CRFK cells) at an MOI of 0.1 and incubated for an additional 48 h. Virus-infected cells treated with remdesivir (at the same concentrations) or DMSO (1% *w/w*) were used as positive and negative controls, respectively. Viral titers were determined from the collected supernatant media by a plaque assay and used to construct dose–response curves (dependence of the viral titers [PFU/mL] on the compound concentration [µM]) and inhibition curves (dependence of the inhibition percentage on the compound concentration [µM]) and for calculation of the 50% effective concentrations (EC_50_; the concentration of compound required to inhibit the viral titer by 50% compared to the control value).

### 2.7. Plaque Assay

Plaque assays were performed using Vero E6 cells (ATCC CRL-1586) as described previously [28,29]. Briefly, 10-fold dilutions of SARS-CoV-2 or FIPV were prepared in 24-well tissue culture plates, and the cells were added to each well (0.6–1.5 × 10^5^ cells per well). After a 4 h incubation at 37 °C and 5% CO_2_, the suspension was overlaid with 1.5% (*w/v*) carboxymethylcellulose in a two-fold concentrated DMEM medium. Following a 5-day incubation at 37 °C and 5% CO_2_, the infected plates were washed with phosphate-buffered saline, and the cell monolayers were stained with naphthalene black. The virus titer was expressed as plaque-forming units (PFU)/mL.

## 3. Results and Discussion

**Scheme 1:**
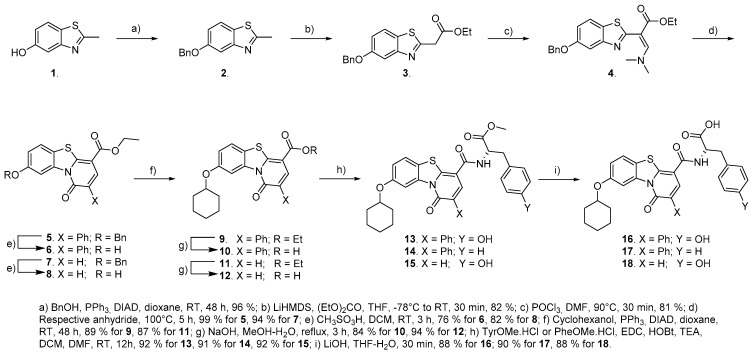
Synthetic pathway to HeE1-2Tyr (**16**) and its derivatives.

Although the synthesis of HeE1-2Tyr (**16**) and its derivatives has been well documented by work of Tarantino et al., we devised a modified approach in order to have easier access to a broader range of substituents in the 8-OH position of the benzothiazolo pyridinone (Scheme 1). To achieve this goal, we decided to protect the 5-OH group of the benzothiazole throughout the stages of central core construction. This way, the alkyl substituent could be introduced by the Mitsunobu reaction just prior to the introduction of the amino acid residue. After initial experiments with a TBDMS, which proved difficult to install and not very stable, we decided to use benzyl for this purpose. The 2-methyl group was then converted to an ethyl acetate by a reaction of an appropriate salt with ethyl carbonate. By using LiHMDS we achieved a very good yield in a short reaction time compared to methods using sodium hydride. A dimethylaminomethylene group was then introduced by the Vilsmeier–Haack reaction, and the pyridinone ring was subsequently closed by heating compound **4** with a neat acyl anhydride—acetic or phenylacetic. The benzyl group was comfortably cleaved using methansulfonic acid, providing compounds suitable for the Mitsunobu reaction, which afforded products **9** and **11**, respectively. Workup of compounds **5**–**8** proved to be very easy, as these compounds are very poorly soluble, and reaction conversions are high. The rest of the synthesis was performed in a similar way as reported by Tarantino et al. An ethyl ester function was saponified, and *L*-Tyrosine and *L*-Phenylalanine methylesters, respectively, were connected to the free carboxylic acid by EDC-HOBt peptide coupling. This set of conditions proved to provide the best yields in the short optimization we performed. This procedure gave HeE1-2Tyr (**16**), as well two novel analogues **17** and **18**, in excellent yields. Detailed descriptions of the synthesis is provided in the Supplementary File.

**Figure 1:**
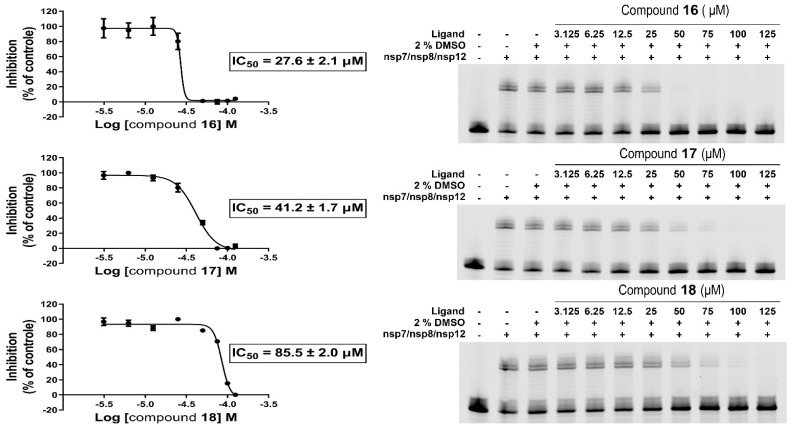
Inhibitions of SARS-CoV-2 RdRp by compounds **16**, **17**, and **18**. (**left**) IC_50_ values were established for each tested compound using a radioactive primer extension assay. Error bars represent the standard error of the mean (*n* = 3). (**right**) A fluorescence-based primer extension assay. Formation of the 40-nt product decreases with increasing concentration of the compound.

Our in vitro RdRp assay confirmed the inhibitory effect of compounds HeE1-2Tyr (**16**), **17**, and **18** against SARS-CoV-2 polymerase. The compounds were initially tested at one concentration (100 µM), which confirmed that compounds HeE1-2Tyr (**16**), **17,** and **18** inhibit SARS-CoV-2 RdRp. Subsequently, the RdRp assay was used to determine the IC_50_ values. Surprisingly, compound HeE1-2Tyr (**16**) was found to be the best inhibitor, with IC_50_ of 27.6 ± 2.1 µM, while the inhibition effect of compound **18** was rather weak (IC_50_ = 85.5 ± 2.0 µM, Figure 1).

**Figure 2:**
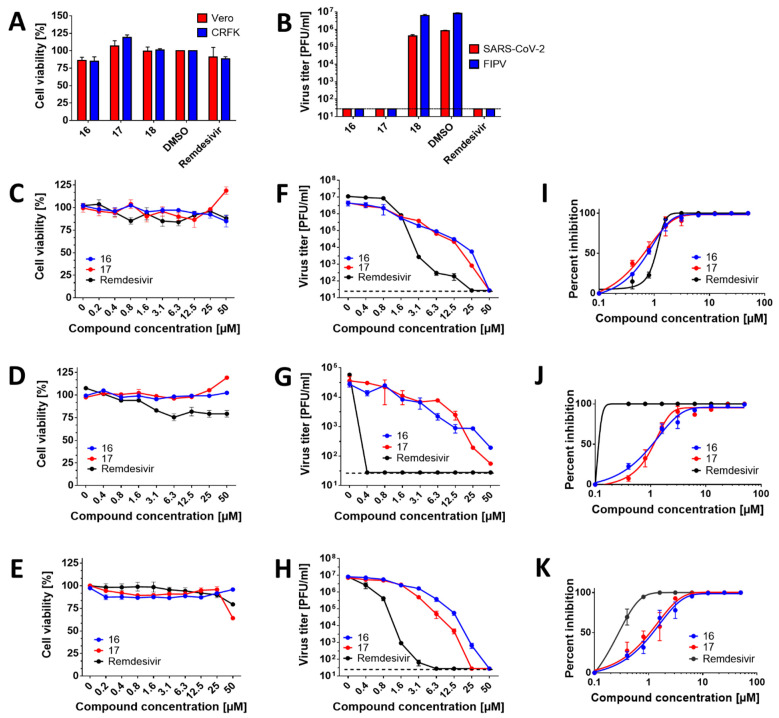
Antiviral efficacy and cytotoxicity of the studied RdRp inhibitors in cell-based assays. (**A**) Cytotoxicity of the indicated compounds in Vero and CRFK cells. The cell monolayers were treated with the compounds (50 µM) and incubated for 48 h. The cell-mediated conversion of tetrazolium salt WST-8 to formazan was monitored by absorbance measurement (450 nm). Cytotoxicity was expressed in terms of cell viability percentage. (**B**) Anti-SARS-CoV-2 and anti-FIPV efficacies of the indicated compounds in Vero and CRFK cells, respectively. The cell monolayers were treated with the compounds (50 µM), infected with the respective virus at a MOI of 0.1, and incubated for 48 h. The supernatant media were collected, and viral titers were determined by plaque assays. (**C**–**E**) Dose-dependent cytotoxic effects of the compounds in Vero (**C**), CaCo-2 (**D**), and CRFK (**E**) cells. The cell monolayers were treated with the compounds in the concentration range from 0 to 50 µM and incubated for 48 h. Cytotoxicity was determined, as described for (A). (**F**,**G**) Dose-dependent anti-SARS-CoV-2 activity of the compounds in Vero (**F**) and CaCo-2 (**G**) cells. The cell monolayers were treated with the compounds in the concentration range 0–50 µM and simultaneously infected with SARS-CoV-2 at a MOI of 0.1. The infected cells were incubated with the compounds for 48 h p.i., and viral titers were determined using the plaque assay. (**H**) Dose-dependent anti-FIPV activity of the compounds in CRFK cells. The protocol was the same as described for (**F**,**G**). (**I**–**K**) Inhibitory curves for the indicated compounds in Vero (**I**), CaCo-2 (**J**), and CRFK (**K**) cells. The mean titers from three biological replicates are shown, and error bars indicate standard errors of the mean (*n* = 3). The horizontal dashed line indicates the minimum detectable threshold of 1.44 log_10_ PFU/mL.

Based on our findings that the compounds showed inhibitory activities in SARS-CoV-2 RdRp assays, we next evaluated cytotoxicity and antiviral activity of HeE1-2Tyr (**16**), **17**, and **18** in cell-based systems. The compounds were initially tested at one single concentration of 50 µM against SARS-CoV-2 and FIPV in Vero and CRFK cells, respectively. This initial screening revealed that all compounds at 50 µM were not cytotoxic for both cell lines (Figure 2A). Moreover, compounds HeE1-2Tyr (**16**) and **17** were found to completely inhibit the replication of both coronaviruses (Figure 2B). Surprisingly, compound **18** was found to be inactive against SARS-CoV-2 and FIPV in both cell-based systems tested (EC_50_ > 50 µM) (Figure 2B). The inactivity could be explained by its poor cellular uptake or extensive degradation by cellular catabolic enzymes. Therefore, compound **18** was excluded from further testing.

The cytotoxicity of compounds HeE1-2Tyr (**16**) and **17** was studied in detail using Vero, CaCo-2, and CRFK cells in a concentration range from 0 to 50 µM. Both compounds showed good cytotoxicity profiles for the studied cell lines and were characterized by CC_50_ values >50 µM (Figure 2C–E; Table 1, Table 2 and Table 3). Interestingly, compound **17** caused a moderate increase in cell viability in Vero and CaCo-2 cells (to approx. 125% of untreated cells) but was slightly cytotoxic for CRFK cells (cell viability of approx. 60%) at the highest concentration tested (50 µM) (Figure 2C–E). The observed changes in the viability of cells treated by compound **17**, however, did not affect the CC_50_ values, which were calculated to be >50 µM for all the cell lines tested.

We further evaluated the dose-dependent, anti-coronaviral activities of compounds HeE1-2Tyr (**16**) and **17**. Anti-SARS-CoV-2 potency of both compounds in Vero cells reached sub-micro molar concentrations, with EC_50_ values of 653.5 nM (for **16**) and 527.3 nM (for **17**). These values were similar or even better than those of remdesivir (EC_50_ = 901.2 nM) (Figure 2F,I, Table 1). Although the EC_50_ values for HeE1-2Tyr (**16**) and **17** were slightly lower compared with those for remdesivir, the growth inhibition curve slopes for remdesivir were substantially steeper than those for HeE1-2Tyr (**16**) and **17**, indicating that remdesivir had a superior inhibitory profile over HeE1-2Tyr (**16**) and **17**. In CaCo-2 cells, compounds HeE1-2Tyr (**16**) and **17** showed EC_50_ values close to 1 µM (949.3 and 995.9 nM, respectively) and had somewhat lower antiviral potencies compared with remdesivir (EC_50_ < 300 nM) (Figure 2G,J, Table 2). Because of their low cytotoxicity, both compounds were characterized by relatively high selectivity indexes (>70 in Vero cells and >50 in CaCo-2 cells) (Table 1 and Table 2). In vitro replication of FIPV, another member of the *Coronaviridae* family used in our antiviral study, was also strongly suppressed with compounds HeE1-2Tyr (**16**) and **17**. The anti-FIPV activity of these compounds was characterized by EC_50_ values of about 1 µM, was approx. 5-fold lower compared with remdesivir (EC_50_ = 223 nM), and showed selectivity indexes exceeding 40 (Figure 2H,K, Table 3).

In conclusion, HeE1-2Tyr (**16**) and its derivatives are potent non-nucleoside inhibitors of coronaviral RdRp that probably act as competitive inhibitors interacting via RNA template tunnel in contrast to chain terminator inhibitors such as remdesivir. These compounds represent suitable candidates for the preparation of clinically usable compounds against SAR-CoV-2 and other coronaviruses, as we have shown with an FIPV example. Compounds HeE1-2Tyr (**16**) and **17** showed significant activity against these coronaviruses in cell cultures. We have also unveiled that one of the mechanisms of action of these compounds is the inhibition of RdRp SARS-CoV-2. This key virus enzyme was inhibited by these compounds, with HeE1-2Tyr (**16**) and **17** showing significantly higher activity compared to **18**. This suggests that the phenyl substituent on the pyridinone portion of the backbone may significantly affect the activity of these derivatives, both in the polymerase assay and in the cell lines. Clearly, further chemical modification and optimization to enhance potency and physicochemical properties will be required for the eventual use of this type of compounds in clinical practice; however, the derivatives of HeE1-2Tyr (**16**) may be one of the rare non-nucleoside inhibitors of coronavirus replication that interfere with RdRp, a key enzyme of these viruses. Although remdesivir is the only RdRp-targeting inhibitor approved by the FDA and EMA for clinical use, some studies suggest that its in vivo effects are suboptimal. Experience with other viral diseases suggests that combinations with other polymerase inhibitors can provide a significant synergistic effect and prevent the evolution of drug-resistant virus mutants; therefore, the introduction of new types of inhibitors may have a significant impact on improving the clinical outcome for patients.

## Figures and Tables

**Table 1:** Anti-SARS-CoV-2 activity and cytotoxicity of the tested compounds in Vero cells.

Compound	EC_50_ (µM) (95% CI) ^a,b^	CC_50_ (µM) ^a^	SI ^c^
**16**	0.6535 (0.4376–0.9760)	>50	>76.5
**17**	0.5273 (0.4423–0.6286)	>50	>94.8
**18**	>50	>50	>1
Remdesivir	0.9012 (0.7997–1.016)	>50	>55.5

[^a^] Determined from three independent experiments. [^b^] Expressed as a 50% reduction in viral titers and calculated as inflection points of sigmoidal inhibitory curves which were obtained by a nonlinear fit of transformed inhibitor concentrations versus normalized response using GraphPad Prism 7.04 (GraphPad Software, Inc., USA). [^c^] CC_50_/EC_50_.

**Table 2:** Anti-SARS-CoV-2 activity and cytotoxicity of the tested compounds in CaCo-2 cells.

Compound	EC_50_ (µM) (95% CI) ^a,b^	CC_50_ (µM) ^a^	SI ^c^
**16**	0.9493 (0.5131–1.756)	>50	>52.7
**17**	0.9959 (0.5427–1.827)	>50	>50.2
**18**	>25	>50	>2
Remdesivir	<0.3	>50	>216.9

For [^a–c^] see Table 1.

**Table 3:** Anti-FIPV activity and cytotoxicity of the tested compounds in CRFK cells.

Compound	EC_50_ (µM) (95% CI) ^a,b^	CC_50_ (µM) ^a^	SI ^c^
**16**	1.062 (0.8188–1.377)	>50	>47.1
**17**	0.9989 (0.8274–1.206)	>50	>50.1
**18**	>50	>50	>1
Remdesivir	0.2230 (0.1694–0.2937)	>50	>224.2

For [^a–c^] see Table 1.

## Data Availability

Not applicable.

## Supplementary Materials

The following are available online at https://www.mdpi.com/article/10.3390/v13081585/s1, Supplementary File S1: chemical synthesis of the compounds and NMR spectra.
