# Supplementary material for: Non-Nucleotide RNA-Dependent RNA Polymerase Inhibitor That Blocks SARS-CoV-2 Replication
Source: Viruses. 2021 Aug 11;13(8):1585. doi: 10.3390/v13081585 (PMC8402726; doi:10.3390/v13081585)

Supplementary Material

Non-nucleotide RNA-dependent RNA polymerase inhibitor that blocks SARS-CoV-2 replication

Milan Dejmek,[a] Eva Konkoľová,[a] Luděk Eyer,[b,c] Petra Straková, [b,c] Pavel Svoboda, [b,c,d] Michal Šála,[a] Kateřina Krejčová,[a] Daniel Růžek,*[b,c] Evzen Boura,*[a] and Radim Nencka*[a]

[a] Dr. M. Dejmek, Dr. E. Konkoľová, Dr. M Šála, Dr. E. Boura, Dr. R. Nencka, Institute of Organic Chemistry and Biochemistry, Czech Academy of Sciences, Flemingovo náměstí 542/2, 160 00 Praha 6, Czech Republic, E-mail: boura@uochb.cas.cz, nencka@uochb.cas.cz

[b] Dr. L Eyer, Dr. P. Straková, Dr. P. Svoboda, Prof. Dr. D. Růžek, Veterinary Research Institute, Emerging Viral Diseases, Hudcova 296/70, 621 00 Brno, Czech Republic, E-mail: ruzekd@paru.cas.cz

[c] Dr. L. Eyer, Dr. P. Straková, Dr. P. Svoboda, Prof. Dr. D. Růžek, Institute of Parasitology, Biology Centre of the Czech Academy of Sciences, Branišovská 1160/31, CZ-37005 České Budějovice, Czech Republic

[d] Dr. P. Svoboda, Department of Pharmacology and Pharmacy, Faculty of Veterinary Medicine, University of Verterinary and Pharmaceutical Sciences Brno, Palackého tř. 1946/1, 612 42 Brno, Czech Republic

Synthesis of inhibitors

Methods and instrumentation

NMR spectra were recorded on Bruker Avance III HD (1H at 400 MHz) spectrometer using DMSO-*d*6 or $CDCl_3$ as a solvent and the solvent signal as a reference. Chemical shifts (*δ*) and coupling constants (J) were expressed in ppm and Hz, respectively. All structures were confirmed and 1H and 13C signals were assigned by a combination of 1D and 2D NMR (H,H-COSY, H,C-HSQC, H,C-HMBC) techniques. Standard pulse programs from the library of the spectrometer were used; gradient selection was used in the 2D experiments. Mass spectra were measured on an LTQ Orbitrap XL using electrospray ionization (ESI). Column chromatography (both normal and reverse phase) was performed on a 40-60 µm silica gel using ISCO flash chromatography system. Purity of all prepared compounds was higher than 95% unless stated otherwise.

NMR data (copies of spectra, lists of multiplets/peaks and their assignment to corresponding atoms) is attached even in cases, where compounds have already been published elsewhere. Such articles are cited in the references section.

5-(benzyloxy)-2-methylbenzo[*d*]thiazole (**2**)

To a vigorously stirred, ice-cooled solution of 2-methylbenzo[*d*]thiazol-5-ol **1** (500 mg, 3 mmol) in anhydrous DMF (5 mL) was added NaH (60 % in mineral oil, 132 mg, 3.3 mmol) in 3 portions. After 30 minutes benzyl bromide (410 µL, 3.45 mmol) was added dropwise at 0 °C, reaction mixture was then allowed to reach ambient temperature and stirred for further 4 hours. After carefully quenching the reaction with sat. aq. NH_4Cl (30 mL) and water (30 mL) the product was extracted with AcOEt (2 x 50 mL). Combined organic fractions were dried with sodium sulfate, evaporated and the product was purified by flash chromatography (AcOEt in cyclohexane, 30 – 70 %) affording **2** (733 mg, 96 %) as pale yellow solid. NMR spectra were consistent with literature[1].

Ethyl 2-(5-(benzyloxy)benzo[*d*]thiazol-2-yl)acetate (**3**)

To a solution of **2** (511 mg, 2 mmol) in anhydrous THF (10 mL) at -78 °C under argon atmosphere, LiHMDS (1M in THF, 4.4 mL, 4.4 mmol) was added dropwise. After 30 minutes diethyl carbonate (291 µL, 2.4 mmol) was added dropwise at -78 °C, reaction mixture was then allowed to reach 0 °C and stirred for further 1 hour. The reaction was poured in 0.2 M HCl (50 mL), product was extracted with AcOEt (2x 50 mL), combined organic layers were successively washed with sat. aq. $NaHCO_3$ (30 mL) and water (30 mL) and then dried with sodium sulfate and evaporated. The product was purified by flash chromatography (AcOEt in cyclohexane, 10 – 50 %) affording **3** (540 mg, 82 %) as a light brown oil, which solidified on standing. ^{1}H NMR (401 MHz, $CDCl_3$) δ 7.72 (dd, $J_{4,5}$ = 8.8, $J_{4,7}$ = 0.5 Hz, 1H, H-4), 7.56 (d, $J_{7,5}$ = 2.4 Hz, 1H, H-7), 7.49 – 7.44 (m, 2H, Bn-*o*), 7.42 – 7.37 (m, 2H, Bn-*m*), 7.35 – 7.30 (m, 1H, Bn-*p*), 7.12 (dd, $J_{5,4}$ = 8.8, $J_{5,7}$ = 2.5 Hz, 1H, H-5), 5.15 (s, 2H, Bn-CH_2), 4.25 (q, $J_{CH2,CH3}$ = 7.1 Hz, 2H, <u>CH_2</u>CH_3), 4.14 (s, 2H, CH_2), 1.30 (t, $J_{CH3,CH2}$ = 7.2 Hz, 3H, CH_2<u>CH_3</u>).

^{13}C NMR (101 MHz, $CDCl_3$) δ 168.59 (COO), 163.96 (C-2), 158.15 (C-6), 154.10 (C-7a), 136.80 (Bn-*i*), 128.78 (Bn-*m*), 128.21 (Bn-*p*), 128.07 (C-4a), 127.66 (Bn-*o*), 121.94 (C-4), 116.21 (C-5), 106.87 (C-7), 70.56 (Bn-CH_2), 61.90 ($\underline{CH_2}CH_3$), 40.03 (CH_2), 14.27 ($CH_2\underline{CH_3}$). ESI MS *m/z* (%): 328.1 (49) [M+H], 350.1 (100) [M+Na]; HRMS ESI ($C_{18}H_{18}O_3NS$) calculated: 498.02874; found: 498.02867.

Ethyl (*Z*)-2-(5-(benzyloxy)benzo[*d*]thiazol-2-yl)-3-(dimethylamino)acrylate (**4**)

$POCl_3$ (284 µL, 3.1 mmol) was dropwise added to an ice-cooled, vigorously stirred DMF (710 µL, 9.2 mmol) and this mixture was stirred at ambient temperature for 30 minutes. To thus formed Vilsmeier reagent was added **3** (500 mg, 1.5 mmol) and the reaction mixture was stirred at 90 °C for 1 hour. Water was added after cooling to ambient temperature, pH was adjusted to ca 8 with solid K_2CO_3 and the product was extracted with AcOEt (3 x 50 mL). Combined organic layers were washed with water and brine, dried with sodium sulfate and evaporated to afford **4** (465 mg, 81 %) as a light brown oil, which was immediately used in the next reaction without further purification. Slow decomposition to appropriate formyl derivative is observed.

Ethyl 8-(benzyloxy)-1-oxo-2-phenyl-1*H*-benzo[4,5]thiazolo[3,2-a]pyridine-4-carboxylate (**5**)

A mixture of **4** (1 g, 2.6 mmol) and phenylacetic anhydride (1.33 g, 5.2 mmol) was heated to 100 °C with stirring. After 15 minutes the reaction mixture solidified, was cooled down, dissolved in $CHCl_3$ (5 mL) and MeOH (100 mL) was added. Precipitated product was collected on a paper filter, washed thoroughly with methanol and dried in high vacuum to afford **5** (1.11 g, 94 %) as light yellow powder. ^1H NMR (401 MHz, $CDCl_3$) δ 9.18 (d, $J_{9,7}$ = 2.5 Hz, 1H, H-9), 8.21 (s, 1H, H-3), 7.77 – 7.71 (m, 2H, Ph-*o*), 7.62 (d, $J_{6,7}$ = 8.7 Hz, 1H, H-6), 7.52 – 7.44 (m, 4H, Bn-*o,m*), 7.43 – 7.31 (m, 4H, Ph-*m,p*, Bn-*p*), 7.19 (dd, $J_{7,6}$ = 8.7, $J_{7,9}$ = 2.5 Hz, 1H, H-7), 5.17 (s, 2H, Bn-CH_2), 4.44 (q, $J_{CH2,CH3}$ = 7.1 Hz, 2H, $\underline{CH_2}CH_3$), 1.43 (t, $J_{CH3,CH2}$ = 7.1 Hz, 3H, $CH_2\underline{CH_3}$). ^{13}C NMR (101 MHz, $CDCl_3$) δ 165.05 (COO), 162.13 (C-1), 158.06 (C-8), 153.47 (C-4a), 139.47 (C-9a), 136.49 and 136.40 (Bn-*i*, Ph-*i*), 135.82 (C-3), 129.01 (Bn-*m*), 128.72 (Ph-*o*), 128.53 (Ph-*m*), 128.24 and 127.87 (Ph-*p*, Bn-*p*), 127.78 (Bn-*o*), 123.96 (C-2), 121.90 (C-6), 119.99 (C-5a), 116.77 (C-7), 105.97 (C-9), 103.26 (C-4), 70.66 (Bn-CH_2), 61.58 ($\underline{CH_2}CH_3$), 14.62 ($CH_2\underline{CH_3}$). ESI MS *m/z* (%): 456.1 (100) [M+H], 478.1 (76) [M+Na]; HRMS ESI ($C_{27}H_{22}O_4NS$) calculated: 456.12641; found: 456.12635.

Ethyl 8-(benzyloxy)-1-oxo-1*H*-benzo[4,5]thiazolo[3,2-a]pyridine-4-carboxylate (**7**)

A solution of **4** (1 g, 2.6 mmol) in acetic anhydride (8 mL) was stirred at reflux temperature for 4 hours. Volatiles were evaporated, the very poorly soluble product was precipitated on addition of methanol (100 mL), collected on a paper filter and dried in high vacuum to afford **7** (990 mg, 99 %) as light yellow powder. ^1H NMR (401 MHz, $CDCl_3$) δ 9.09 (d, $J_{9,7}$ = 2.4 Hz, 1H, H-9), 8.04 (d, $J_{3,2}$ = 9.5 Hz, 1H, H-3), 7.61 (d, $J_{6,7}$ = 8.7 Hz, 1H, H-6), 7.53 – 7.47 (m, 2H, Bn-*o*), 7.44 – 7.38 (m, 2H, Bn-*m*), 7.38 – 7.30 (m, 1H, Bn-*p*), 7.18 (dd, $J_{7,6}$ = 8.7, $J_{7,9}$ =2.5 Hz, 1H, H-7), 6.44 (d, $J_{2,3}$ = 9.4 Hz, 1H, H-2), 5.20 (s, 2H, Bn-CH_2), 4.41 (q, J_{CH2CH3} = 7.1 Hz, 2H, $\underline{CH_2}CH_3$), 1.42 (t, J_{CH3CH2} = 7.1 Hz, 3H, $CH_2\underline{CH_3}$). ^{13}C NMR (101 MHz, $CDCl_3$) δ 164.88 (COO), 162.93 (C-1), 157.99 (C-8), 154.90 (C-4a), 139.08 (C-9a), 137.35 (C-3), 136.50 (Bn-*i*), 128.77 (Bn-*m*), 128.29 (Bn-*p*), 127.84 (Bn-*o*), 121.80 (C-6), 119.67 (C-5a), 116.50 (C-7), 112.34 (C-2), 106.00 (C-9), 103.24 (C-4), 70.69 (Bn-CH_2), 61.48 ($\underline{CH_2}CH_3$), 14.57 ($CH_2\underline{CH_3}$). ESI MS *m/z* (%): 380.1 (74) [M+H], 402.1 (100) [M+Na]; HRMS ESI ($C_{21}H_{17}O_4NNaS$) calculated: 402.07705; found: 402.07718.

Ethyl 8-hydroxy-1-oxo-2-phenyl-1*H*-benzo[4,5]thiazolo[3,2-a]pyridine-4-carboxylate (**6**)

To a solution of **5** (1.11 g, 2.44 mmol) in DCM (30 mL) was added methanesulfonic acid (3.17 mL, 48.8 mmol) and the reaction mixture was stirred at ambient temperature for 3 hours. Volatiles were evaporated, residue dissolved in AcOEt (100 mL), washed with sat. soln. of $NaHCO_3$ (50 mL) and brine (50 mL), dried with sodium sulfate and evaporated. Residue was dissolved in chloroform and the product was precipitated by addition of methanol, collected on a paper filter and dried in high vacuum to afford **6** (730 mg, 82 %) as a yellow solid very poorly soluble in polar solvents (DMSO, MeOH). ^1H NMR (401 MHz, $\underline{CDCl_3}$/MeOD) δ 8.75 (d, $J_{9,7}$ = 2.3 Hz, 1H, H-9), 8.19 (s, 1H, H-3), 7.69 – 7.63 (m, 2H, Ph-*o*), 7.58 (d, $J_{6,7}$ = 8.6 Hz, 1H, H-6), 7.47 – 7.41 (m, 2H, Ph-*m*), 7.38 – 7.31 (m, 1H, Ph-*p*), 7.05 (dd, $J_{7,6}$ = 8.6, $J_{7,9}$ = 2.4 Hz, 1H, H-7), 4.41 (q, $J_{CH2,CH3}$ = 7.1 Hz, 2H, $\underline{CH_2}CH_3$), 1.41 (t, $J_{CH3,CH2}$ = 7.1 Hz, 3H, $CH_2\underline{CH_3}$). ^{13}C NMR (101 MHz, $\underline{CDCl_3}$/MeOD) δ 164.92 (COO), 162.09 (C-1), 156.42 (C-8), 153.46 (C-4a), 138.88 (C-9a), 136.07 (C-3), 136.05 (Ph-*i*), 128.77 (Ph-*o*), 128.18 (Ph-*m*), 127.64 (Ph-*p*), 123.49 (C-2), 121.90 (C-6), 118.12 (C-5a), 115.99 (C-7), 107.17 (C-9), 103.37 (C-4), 61.47

($\underline{CH_2}CH_3$), 14.14 ($CH_2\underline{CH_3}$). ESI MS *m/z* (%): 366.1 (65) [M+H], 388.1 (100) [M+Na]; HRMS ESI ($C_{20}H_{16}O_4NS$) calculated: 366.07946; found: 366.07978.

Ethyl 8-hydroxy-1-oxo-1*H*-benzo[4,5]thiazolo[3,2-a]pyridine-4-carboxylate (**8**)

To a solution of **7** (900 mg, 2.35 mmol) in DCM (30 mL) was added methanesulfonic acid (3.06 mL, 47 mmol) and the reaction mixture was stirred at ambient temperature for 3 hours. Volatiles were evaporated and the residue was separated between $CHCl_3$ (100 mL) and $NaHCO_3$ (60 mL). The very poorly soluble product was extracted from the aqueous layer with further portions of $CHCl_3$ (5 x 50 mL), pooled organic layers were dried with sodium sulfate and evaporated. Product was susoended in AcOEt (50 mL), sonicated for 10 minutes and collected on a paper filter to afford **8** (518 mg, 76 %) as a yellow solid. ^1H NMR (401 MHz, DMSO-d_6) δ 10.05 (s, 1H, OH), 8.68 (d, $J_{9,7}$ = 2.3 Hz, 1H, H-9), 7.96 (d, $J_{3,2}$ = 9.5 Hz, 1H, H-3), 7.85 (d, $J_{6,7}$ = 8.6 Hz, 1H, H-6'), 7.02 (dd, $J_{7,6}$ = 8.6, $J_{7,9}$ = 2.4 Hz, 1H, H-7), 6.35 (d, $J_{2,3}$ = 9.4 Hz, 1H, H-2), 4.31 (q, J_{CH2CH3} = 7.1 Hz, 2H, $\underline{CH_2}CH_3$), 1.33 (t, J_{CH3CH2} = 7.1 Hz, 3H, $CH_2\underline{CH_3}$). ^{13}C NMR (101 MHz, DMSO) δ 164.14 (COO), 161.84 (C-1), 156.73 (C-8), 154.93 (C-4a), 138.55 (C-9a), 137.23 (C-3), 122.97 (C-6), 117.14 (C-5a), 115.75 (C-7), 111.77 (C-2), 106.40 (C-9), 102.16 (C-4), 61.12 ($\underline{CH_2}CH_3$), 14.42 ($CH_2\underline{CH_3}$). negESI MS *m/z* (%): 288.0 (100) [M-H]; HRMS ESI ($C_{14}H_{10}O_4NS$) calculated: 288.03360; found: 288.03384.

Ethyl 8-(cyclohexyloxy)-1-oxo-2-phenyl-1*H*-benzo[4,5]thiazolo[3,2-a]pyridine-4-carboxylate (**9**)

To a stirred suspension of **6** (600 mg, 1.64 mmol) in anhydrous dioxane (15 mL) were sequentially added PPh_3 (860 mg, 3.28 mmol), cyclohexanol (340 µL, 3.28 mmol) and DIAD (640 µL, 3.28 mmol). On addition of DIAD the starting material started rapidly dissolving, stirring was continued for 1 hour. Volatiles were evaporated and flash chromatography of the residue (AcOEt in cyclohexane, 0-50 %) afforded **9** (640 mg, 87 %) as light yellow solid. NMR spectra were consistent with literature.[2] ^1H NMR (401 MHz, $CDCl_3$) δ 9.06 (d, $J_{9,7}$ = 2.4 Hz, 1H, H-9), 8.20 (s, 1H, H-3), 7.75 – 7.71 (m, 2H, Ph-*o*), 7.61 (d, $J_{6,7}$ = 8.7 Hz, 1H, H-6), 7.50 – 7.44 (m, 2H, Ph-*m*), 7.40 – 7.35 (m, 1H, Ph-*p*), 7.12 (dd, $J_{7,6}$ = 8.7, $J_{7,9}$ = 2.4 Hz, 1H, H-7), 4.47 - 4.40 (m, 1H, H-1'), 4.44 (q, $J_{CH2,CH3}$ = 7.1 Hz, 2H, OCH_2), 2.03 – 1.95 (m, 2H, H-2'a), 1.84 – 1.75 (m, 2H, H-3'a), 1.64 – 1.52 (m, 4H, H-2'b, 3'b), 1.45 – 1.32 (m, 2H, H-4'), 1.43 (t, $J_{CH3,CH2}$ = 7.1 Hz, 3H, OCH_3). ESI MS *m/z* (%): 448.2 (100) [M+H], 470.2 (61) [M+Na]; HRMS ESI ($C_{26}H_{25}O_4NS$) calculated: 448.15826; found: 448.15833.

Ethyl 8-(cyclohexyloxy)-1-oxo-1*H*-benzo[4,5]thiazolo[3,2-a]pyridine-4-carboxylate (**11**)

To a stirred suspension of **8** (290 mg, 1 mmol) in anhydrous dioxane (10 mL) were sequentially added PPh_3 (525 mg, 2 mmol), cyclohexanol (200 mg, 2 mmol) and DIAD (394 µL, 2 mmol). On addition of DIAD the starting material started rapidly dissolving, stirring was continued for 1 hour. Volatiles were evaporated and flash chromatography of the residue (AcOEt in cyclohexane, 20-50 %) afforded **11** (331 mg, 89 %) as light yellow solid. NMR spectra were consistent with literature.[2] ^1H NMR (401 MHz, $CDCl_3$) δ 8.97 (d, $J_{9,7}$ = 2.4 Hz, 1H, H-9), 8.03 (d, $J_{3,2}$ = 9.4 Hz, 1H, H-3), 7.58 (d, $J_{6,7}$ = 8.7 Hz, 1H, H-6'), 7.09 (dd, $J_{7,6}$ = 8.7, $J_{7,9}$ = 2.4 Hz, 1H, H-7), 6.42 (d, $J_{2,3}$ = 9.5 Hz, 1H, H-2), 4.44 – 4.36 (m, H, H-1'), 4.40 (q, J_{CH2CH3} = 7.1 Hz, 2H, $\underline{CH_2}CH_3$), 2.08 – 1.98 (m, 2H, H-2'a), 1.86 – 1.77 (m, 2H, H-3'a), 1.64 – 1.51 (m, 3H, H-2'b, H-3'b). 1.46 – 1.28 (m, 3H, H-3'b, H-4'), 1.41 (t, J_{CH3CH2} = 7.1 Hz, 3H, $CH_2\underline{CH_3}$). ESI MS *m/z* (%): 372.0 (100) [M+H], 394.0 (76) [M+Na]; HRMS ESI ($C_{20}H_{22}O_4NS$) calculated: 372.12641; found: 372.12650.

8-(cyclohexyloxy)-1-oxo-2-phenyl-1*H*-benzo[4,5]thiazolo[3,2-a]pyridine-4-carboxylic acid (**10**)

To a solution of **9** (230 mg, 0.5 mmol) in methanol (10 mL) was added aqueous NaOH (2M, 3 mL) and reaction mixture was stirred at reflux temperature for 2 hours. After cooling down pH was adjusted to ca 1 with 1N HCl, product was extracted with $CHCl_3$ (3 x 25 mL), pooled organic phases were dried with sodium sulfate and evaporated. Residual solid was dissolved in a small amount of DCM and product was precipitated with methanol to afford **10** (203 mg, 94%) as light yellow solid. NMR spectra were consistent with literature.[2] ^1H NMR (401 MHz, $CDCl_3$) δ 9.03 (d, $J_{9,7}$ = 2.4 Hz, 1H, H-9), 8.26 (s, 1H, H-3), 7.75 – 7.69 (m, 2H, Ph-*o*), 7.65 (d, $J_{6,7}$ = 8.6 Hz, 1H, H-6), 7.50 – 7.41 (m, 3H, Ph-*m*, H-7), 7.40 – 7.33 (m, 1H, Ph-*p*), 4.47 - 4.38 (m, 1H, H-1'), 2.05 – 1.95 (m, 2H, H-2'a), 1.85 – 1.75 (m, 2H, H-3'a), 1.63 – 1.52 (m, 3H, H-2'b, 3'b), 1.48 – 1.30 (m, 3H, 3'b, H-4'). NegESI MS *m/z* (%): 418.1 (100) [M-H]; HRMS ESI ($C_{24}H_{21}O_4NS$) calculated: 418.11131; found: 418.11099.

8-(cyclohexyloxy)-1-oxo-1*H*-benzo[4,5]thiazolo[3,2-a]pyridine-4-carboxylic acid (**12**)

To a solution of **11** (300 mg, 0.81 mmol) in methanol (20 mL) was added aqueous NaOH (10 %, 5 mL) and reaction mixture was stirred at reflux temperature for 2 hours. After cooling down pH was adjusted to ca 1 with 1N HCl, extracted with $CHCl_3$ (3x 25 mL), pooled organic phases were dried with sodium sulfate and evaporated. Residual solid was dissolved in a small amount of DCM and product was precipitated with methanol to afford **12** (233 mg, 84%) as light yellow solid of very poor solubility. NMR spectra were consistent with literature.[2] ^1H NMR (400 MHz, DMSO-d_6) δ 13.20 (bs, 1H, COOH), 8.78 (d, $J_{9,7}$ = 2.4 Hz, 1H, H-9), 8.00 (d, $J_{3,2}$ = 9.4 Hz, 1H, H-3), 7.94 (d, $J_{6,7}$ = 8.8 Hz, 1H, H-6), 7.22 (dd, $J_{7,6}$ = 8.8, $J_{7,9}$ = 2.5 Hz, 1H, H-7), 4.40 (tt, $J_{1',2'a}$ = 8.6, $J_{1',2'b}$ = 3.7 Hz, 1H, H-1'), 2.00 – 1.93 (m, 2H, H-2'a), 1.78 – 1.70 (m, 2H, H-3'a), 1.58 – 1.17 (m, 6H, H-2'b, H-3'b, H-4'). ^{13}C NMR (101 MHz, DMSO-d_6) δ 166.08 (COO), 162.10 (C-1), 156.30 (C-8), 154.78 (C-4a), 138.70 (C-9a), 137.96 (C-3), 123.07 (C-6), 119.41 (C-5a), 116.08 (C-7), 111.76 (C-2), 106.95 (C-9), 103.21 (C-4), 75.53 (C-1'), 31.33 (C-2'), 25.27 (C-4'), 23.29 (C-3'). NegESI MS *m/z* (%): 342.1 (100) [M-H]; HRMS ESI ($C_{18}H_{16}O_4NS$) calculated: 342.08055; found: 342.08008.

Methyl (8-(cyclohexyloxy)-1-oxo-2-phenyl-1*H*-benzo[4,5]thiazolo[3,2-a]pyridine-4-carbonyl)-*L*-tyrosinate (**13**)

To a solution of **10** (90 mg, 0.21 mmol) and *L*-tyrosine methyl ester (50 mg, 0.26 mmol) in DCM-DMF mixture (1:1, 4 mL) were sequentially added HOBt (35 mg, 0.26 mmol), TEA (72 µL, 0.52 mmol) and EDCI (49 mg, 0.26 mmol) and the reaction mixture was stirred at ambient temperature for 12 hours. Volatiles were evaporated and product was isolated by flash chromatography (AcOEt in cyclohexane, 5-60%) to afford **13** (118 mg, 92 %) as light yellow solid. NMR spectra were consistent with literature.[3] ^1H NMR (401 MHz, DMSO-d_6) δ 9.21 (s, 1H, OH), 8.97 (d, $J_{NH,\alpha}$ = 7.7 Hz, 1H, NH), 8.87 (d, $J_{9,7}$ = 2.4 Hz, 1H, H-9), 8.49 (s, 1H, H-3), 7.90 (d, $J_{6,7}$ = 8.7 Hz, 1H, H-6), 7.82 – 7.77 (m, 2H, Ph-*o*), 7.53 – 7.47 (m, 2H, Ph-*m*), 7.42 – 7.36 (m, 1H, Ph-*p*), 7.21 (dd, $J_{7,6}$ = 8.8, $J_{7,9}$ = 2.4 Hz, 1H, H-7), 7.10 – 7.05 (m, 2H, Tyr-2), 6.67 – 6.62 (m, 2H, Tyr-3), 4.64 (ddd, $J_{\alpha,\beta2}$ = 9.6, $J_{\alpha,NH}$ = 7.6, $J_{\alpha,\beta1}$ = 5.8 Hz, 1H, H-α), 4.41 (tt, $J_{1',2'a}$ = 8.4, $J_{1',2'b}$ = 3.7 Hz, 1H, H-1'), 3.64 (s, 3H, OCH_3), 3.06 (dd, J_{GEM} = 13.8, $J_{\beta1,\alpha}$ = 5.8 Hz, 1H, H-β1), 2.98 (dd, J_{GEM} = 13.8, $J_{\beta2,\alpha}$ = 9.6 Hz, 1H, H-β2), 1.99 – 1.91 (m, 2H, H-2'a), 1.77 – 1.68 (m, 2H, H-3'a), 1.57 – 1.26 (m, 6H, H-2'b, 3'b, H-4'). ^{13}C NMR (101 MHz, DMSO-d_6) δ 172.53 (COO), 164.31 (CON), 161.07 (C-1), 156.16 and 156.12 (Tyr-4, C-8), 151.81 (C-4a), 138.74 (C-9a), 136.51 (Ph-*i*), 133.69 (C-3), 130.18 (Tyr-2), 129.11 (Ph-*o*), 128.23 (Ph-*m*), 127.66 (Tyr-1), 127.60 (Ph-*p*), 122.90 (C-6), 122.19 (C-2), 120.61 (C-5a), 116.31 (C-7), 107.33 (C-9), 104.90 (C-4), 75.47 (C-1'), 54.93 (C-α), 52.15 (OCH_3), 35.91 (C-β), 31.33 (C-2'), 25.27 (C-4'), 23.22 (C-3'). ESI MS *m/z* (%): 597.3 (100) [M+H], 619.3 (91) [M+Na]; HRMS ESI ($C_{34}H_{32}O_6N_2S$) calculated: 597.20593; found: 597.20589.

Methyl (8-(cyclohexyloxy)-1-oxo-2-phenyl-1*H*-benzo[4,5]thiazolo[3,2-a]pyridine-4-carbonyl)-*L*-phenylalaninate (**14**)

To a solution of **10** (90 mg, 0.21 mmol) and *L*-phenylalanine methyl ester (56 mg, 0.26 mmol) in DCM-DMF mixture (1:1, 4 mL) were sequentially added HOBt (35 mg, 0.26 mmol), TEA (118 µL, 0.78 mmol) and EDCI (49 mg, 0.26 mmol) and the reaction mixture was stirred at ambient temperature for 12 hours. Volatiles were evaporated and product was isolated by flash chromatography (AcOEt in cyclohexane, 5-60%) to afford **14** (113 mg, 91 %) as light yellow solid. ^1H NMR (401 MHz, $CDCl_3$) δ 8.78 (d, $J_{9,7}$ = 2.4 Hz, 1H, H-9), 7.70 – 7.67 (m, 2H, Ph-*o*), 7.67 (s, 1H, H-3), 7.51 (d, $J_{6,7}$ = 8.7 Hz, 1H, H-6), 7.49 – 7.44 (m, 2H, Ph-*m*), 7.41 – 7.34 (m, 1H, Ph-*p*), 7.34 – 7.22 (m, 2H, Phe-*m*), 7.21 – 7.15 (m, 3H, Phe-*p*, Phe-*o*), 6.99 (dd, $J_{7,6}$ = 8.7, $J_{7,9}$ = 2.4 Hz, 1H, H-7), 5.06 (ddd, $J_{\alpha,NH}$ = 7.5, $J_{\alpha,\beta1}$ = 6.8, $J_{\alpha,\beta2}$ = 5.8 Hz, 1H, H-α), 4.34 (tt, $J_{1',2'a}$ = 8.3, $J_{1',2'b}$ = 3.7 Hz, 1H, H-1'), 3.68 (s, 3H, OCH_3), 3.27 (dd, J_{GEM} = 13.8, $J_{\beta2,\alpha}$ = 5.9 Hz, 1H, H-β2), 3.19 (dd, J_{GEM} = 13.8, $J_{\beta1,\alpha}$ = 6.8 Hz, 1H, H-β1), 2.02 – 1.90 (m, 2H, H-2'b), 1.84 – 1.72 (m, 2H, H-3'b), 1.64 – 1.47 (m, 3H, H-2'a, 4'b), 1.46 – 1.30 (m, 3H, H-3'a, H-4'a). ^{13}C NMR (101 MHz, $CDCl_3$) δ 173.65 (COO), 164.35 (CON), 161.70 (C-1), 156.82 (C-8), 152.23 (C-4a), 138.77 (C-9a), 136.72 (Ph-*i*), 136.04 (Phe-*i*), 132.34 (C-3), 129.32 (Phe-*o*), 129.20 (Ph-*o*), 128.83 (Ph-*m*), 128.50 (Phe-*m*), 127.79 and 127.40 (Ph-*p*, Phe-*p*), 123.92 (C-2), 121.71 (C-6), 120.14 (C-5a), 117.41 (C-7), 107.17 (C-9), 104.53 (C-4), 75.80 (C-1'), 54.18 (C-α), 52.76 (OCH_3), 38.12 (C-β), 31.83 and 31.58 (C-2'), 25.75 (C-4'), 23.65 and 23.57 (C-3'). ESI MS *m/z* (%): 581.2 (100) [M+H], 603.2 (88) [M+Na]; HRMS ESI ($C_{34}H_{33}O_5N_2S$) calculated: 581.21047; found: 581.21032.

Methyl (8-(cyclohexyloxy)-1-oxo-1*H*-benzo[4,5]thiazolo[3,2-a]pyridine-4-carbonyl)-*L*-tyrosinate (**15**)

To a solution of **12** (90 mg, 0.21 mmol) and *L*-tyrosine methyl ester (50 mg, 0.26 mmol) in DCM-DMF mixture (1:1, 4 mL) were sequentially added HOBt (35 mg, 0.26 mmol), TEA (72 µL, 0.52 mmol) and EDCI (49 mg, 0.26 mmol) and the reaction mixture was stirred at ambient temperature for 12 hours. Volatiles were evaporated and product was isolated by flash chromatography (AcOEt in cyclohexane, 5-30%) to afford **9** (118 mg, 92 %) as light yellow solid. ^1H NMR (401 MHz, $CDCl_3$) δ 8.86 (d, $J_{9,7}$ = 2.4 Hz, 1H, H-9), 7.57 (d, $J_{3,2}$ = 9.5 Hz, 1H, H-3), 7.53 (d, $J_{6,7}$ = 8.7 Hz, 1H, H-6), 7.05 (dd, $J_{7,6}$ = 8.7, $J_{7,9}$ = 2.4 Hz, 1H, H-7), 6.99 – 6.93 (m, 2H, Tyr-2),

6.80 – 6.74 (m, 3H, Tyr-3, OH), 6.73 (d, $J_{NH,\alpha}$ = 7.7 Hz, 1H, NH), 6.37 (d, $J_{2,3}$ = 9.4 Hz, 1H, H-2), 5.03 (dt, $J_{\alpha,NH}$ = 7.7, $J_{\alpha,\beta}$ = 5.7 Hz, 1H, H-α), 4.34 (tt, $J_{1',2'a}$ = 8.6, $J_{1',2'b}$ = 4.3 Hz, 1H, H-1'), 3.78 (s, 3H, OCH_3), 3.19 (dd, J_{GEM} = 14.1, $J_{\beta1,\alpha}$ = 5.7 Hz, 1H, H-β1), 3.13 (dd, J_{GEM} = 14.1, $J_{\beta2,\alpha}$ = 5.9 Hz, 1H, H-β2), 2.04 – 1.95 (m, 2H, H-2'a), 1.84 – 1.73 (m, 2H, H-3'a), 1.61 – 1.49 (m, 3H, H-2'b, 3'b), 1.45 – 1.30 (m, 3H, 3'b, H-4'). ^{13}C NMR (101 MHz, $CDCl_3$) δ 172.83 (COO), 164.00 (CON), 162.96 (C-1), 156.99 and 155.74 (Tyr-4, C-8), 153.79 (C-4a), 138.53 (C-9a), 133.89 (C-3), 130.49 (Tyr-2), 127.11 (Tyr-1), 121.70 (C-6), 120.02 (C-5a), 117.46 (C-7), 115.83 (Tyr-3), 112.00 (C-2), 107.25 (C-9), 105.04 (C-4), 76.34 (C-1'), 53.87 (C-α), 52.77 (OCH_3), 37.14 (C-β), 31.79 and 31.72 (C-2'), 25.71 (C-4'), 23.77 and 23.73 (C-3'). ESI MS *m/z* (%): 521.2 (100) [M+H], 543.2 (70) [M+Na]; HRMS ESI ($C_{28}H_{28}O_6N_2S$) calculated: 521.17463; found: 521.17441.

(8-(cyclohexyloxy)-1-oxo-2-phenyl-1*H*-benzo[4,5]thiazolo[3,2-a]pyridine-4-carbonyl)-*L*-tyrosine (**16**)

To a solution of **15** (100 mg, 0.17 mmol) in dioxane (2.5 mL) was added aqueous LiOH (1M, 0.5 mL) and the reaction mixture was stirred at ambient temperature for 30 minutes. pH was adjusted to ca 2 with 1N HCl and the product was extracted with DCM (3x 10 mL). Product was isolated on RP FCC (ACN in H_2O, 10-100 %), dissolved in a small amount of DCM and finally precipitated with ether to afford **16** (87 mg, 88 %) as light yellow powder. NMR spectra were consistent with literature.[3] ^1H NMR (401 MHz, DMSO-d_6) δ 12.77 (bs, 1H, COOH), 9.18 (bs, 1H, OH), 8.89 - 8.84 (m, 2H, NH, H-9), 8.50 (s, 1H, H-3), 7.89 (d, $J_{6,7}$ = 8.7 Hz, 1H, H-6), 7.82 – 7.78 (m, 2H, Ph-*o*), 7.53 – 7.47 (m, 2H, Ph-*m*), 7.42 – 7.37 (m, 1H, Ph-*p*), 7.20 (dd, $J_{7,6}$ = 8.8, $J_{7,9}$ = 2.4 Hz, 1H, H-7), 7.13 – 7.08 (m, 2H, Tyr-2), 6.66 – 6.62 (m, 2H, Tyr-3), 4.60 (ddd, $J_{\alpha,\beta2}$ = 10.3, $J_{\alpha,NH}$ = 8.1, $J_{\alpha,\beta1}$ = 4.8 Hz, 1H, H-α), 4.40 (tt, $J_{1',2'a}$ = 8.4, $J_{1',2'b}$ = 3.7 Hz, 1H, H-1'), 3.09 (dd, J_{GEM} = 13.9, $J_{\beta1,\alpha}$ = 4.8 Hz, 1H, H-β1), 2.95 (dd, J_{GEM} = 13.9, $J_{\beta2,\alpha}$ = 10.3 Hz, 1H, H-β2), 2.00 – 1.90 (m, 2H, H-2'a), 1.77 – 1.67 (m, 2H, H-3'a), 1.57 – 1.25 (m, 6H, H-2'b, 3'b, H-4'). ^{13}C NMR (101 MHz, DMSO-d_6) δ 173.53 (COO), 164.26 (CON), 161.08 (C-1), 156.09 and 156.05 (Tyr-4, C-8), 151.64 (C-4a), 138.73 (C-9a), 136.54 (Ph-*i*), 133.68 (C-3), 130.16 (Tyr-2), 129.12 (Ph-*o*), 128.22 (Ph-*m*), 128.24 (Tyr-1), 127.58 (Ph-*p*), 122.86 (C-6), 122.16 (C-2), 120.67 (C-5a), 116.28 (C-7), 115.22 (Tyr-3), 107.34 (C-9), 105.15 (C-4), 75.47 (C-1'), 54.88 (C-α), 35.88 (C-β), 31.34 (C-2'), 25.28 (C-4'), 23.23 (C-3'). ESI MS *m/z* (%): 583.3 (100) [M+H], 605.3 (76) [M+Na]; HRMS ESI ($C_{33}H_{30}O_6N_2S$) calculated: 583.19028; found: 583.19001.

(8-(cyclohexyloxy)-1-oxo-2-phenyl-1*H*-benzo[4,5]thiazolo[3,2-a]pyridine-4-carbonyl)-*L*-phenylalanine (**17**)

To a solution of **14** (100 mg, 0.17 mmol) in dioxane (2.5 mL) was added aqueous LiOH (1M, 0.5 mL) and the reaction mixture was stirred at ambient temperature for 30 minutes. pH was adjusted to ca 2 with 1N HCl and the product was extracted with DCM (3x 10 mL). Product was isolated on RP FCC (ACN in H_2O, 10-100 %), dissolved in a small amount of DCM and finally precipitated with ether to afford **17** (88 mg, 90 %) as light yellow powder. ^1H NMR (400 MHz, DMSO-d_6) δ 8.84 (d, $J_{9,7}$ = 2.4 Hz, 1H, H-9), 7.90 – 7.76 (m, 3H, H-6, Ph-*o*), 7.52 - 7.42 (m, 2H, Ph-*m*), 7.38 – 7.28 (m, 3H, Ph-*p*, Phe-*o*), 7.24 – 7.16 (m, 2H, Phe-*m*), 7.14 (dd, $J_{7,6}$ = 8.8, $J_{7,9}$ = 2.4 Hz, 1H, H-7), 7.11 – 7.02 (m, 1H, Phe-*p*), 4.72 – 4.61 (m, 1H, H-α), 4.38 (tt, $J_{1',2'a}$ = 8.3, $J_{1',2'b}$ = 4.1 Hz, 1H, H-1'), 3.34 (dm, J_{GEM} = 13.6 Hz, 1H, H-β2), 3.04 (dm, J_{GEM} = 13.6 Hz, 1H, H-β1), 2.00 – 1.87 (m, 2H, H-2'b), 1.79 – 1.66 (m, 2H, H-3'b), 1.55 – 1.25 (m, 6H, H-2'a, H-3'a, H-4'). ^{13}C NMR (101 MHz, DMSO-d_6) δ 163.75 (CON), 161.06 (COO), 155.90 (C-8), 150.79 (C-4a), 138.64 (C-9a), 136.61 (Ph-*i*, Phe-*i*), 129.22 and 129.19 (Ph-*o*, Phe-*o*), 128.10 (Ph-*m*, Phe-*m*), 127.30 (Ph-*p*), 125.93 (Phe-*p*), 122.63 (C-6), 121.93 (C-2), 120.90 (C-5a), 116.03 (C-7), 107.26 (C-9), 106.17 (C-4), 75.39 (C-1'), 56.18 (C-α), 37.61 (C-β), 31.35 and 31.33 (C-2'), 25.28 (C-4'), 23.23 (C-3'). ESI MS *m/z* (%): 567.2 (100) [M+H], 589.2 (24) [M+Na]; HRMS ESI ($C_{33}H_{31}O_5N_2S$) calculated: 567.19482; found: 567.19479.

(8-(cyclohexyloxy)-1-oxo-1*H*-benzo[4,5]thiazolo[3,2-a]pyridine-4-carbonyl)-L-tyrosine (**18**)

To a solution of **15** (100 mg, 0.17 mmol) in dioxane (2.5 mL) was added aqueous LiOH (1M, 0.5 mL) and the reaction mixture was stirred at ambient temperature for 30 minutes. pH was adjusted to ca 2 with 1N HCl and the product was extracted with DCM (3x 10 mL). Product was isolated on RP FCC (ACN in H_2O, 10-100 %) and finally precipitated with ether from a small amount DCM to afford **18** (87 mg, 88 %) as light yellow powder. ^1H NMR (401 MHz, DMSO-d_6) δ 12.77 (bs, 1H, COOH), 9.18 (bs, 1H, OH), 8.80 (d, $J_{9,7}$ = 2.4 Hz, 1H, H-9), 8.68 (d, $J_{NH,\alpha}$ = 8.17 Hz, 1H, NH), 8.29 (d, $J_{3,2}$ = 9.6 Hz, 1H, H-3), 7.86 (d, $J_{6,7}$ = 8.8 Hz, 1H, H-6), 7.18 (dd, $J_{7,6}$ = 8.8, $J_{7,9}$ = 2.4 Hz, 1H, H-7), 7.14 – 7.08 (m, 2H, Tyr-2), 6.67 – 6.62 (m, 2H, Tyr-3), 6.42 (d, $J_{2,3}$ = 9.5 Hz, 1H, H-2), 4.54 (ddd, $J_{\alpha,\beta2}$ = 10.5, $J_{\alpha,NH}$ = 8.0, $J_{\alpha,\beta1}$ = 4.5 Hz, 1H, H-α), 4.28 (tt, $J_{1',2'a}$ = 8.6, $J_{1',2'b}$ = 3.7 Hz, 1H, H-1'), 3.08 (dd, J_{GEM} = 13.9, $J_{\beta1,\alpha}$ = 4.5 Hz, 1H, H-β1), 2.95 (dd, J_{GEM} = 13.9, $J_{\beta2,\alpha}$ = 10.5 Hz, 1H, H-β2), 2.00 – 1.90 (m, 2H, H-2'a), 1.77 – 1.68 (m, 2H, H-3'a), 1.58 – 1.25 (m, 6H, H-2'b, 3'b, H-4'). ^{13}C NMR (101 MHz, DMSO-d_6) δ 173.53 (COO), 164.15 (CON), 161.94 (C-1), 156.06 and 156.03 (Tyr-4, C-8), 152.90 (C-4a), 138.35 (C-9a),

135.47 (C-3), 130.15 (Tyr-2), 128.27 (Tyr-1), 122.73 (C-6), 120.27 (C-5a), 115.92 (C-7), 115.21 (Tyr-3), 111.41 (C-2), 107.02 (C-9), 105.05 (C-4), 75.50 (C-1'), 54.97 (C-α), 35.71 (C-β), 31.35 (C-2'), 25.27 (C-4'), 23.29 (C-3'). ESI MS *m/z* (%): 507.2 (100) [M+H], 529.1 (22) [M+Na]; HRMS ESI ($C_{27}H_{27}O_6N_2S$) calculated: 507.15843; found: 507.15846.

Protein expression and purification

The SARS-CoV-2 nsp7 (GeneBank: YP_009725303), nsp8 (GeneBank: YP_009725304) and nsp12 (GeneBank: YP_009725307) genes were commercially synthesized as codon-optimized for *E. coli* (Invitrogen). The gene for nsp7 was cloned into a modified pRSF-Duet vector containing N-terminal 6×His tag, followed by a GB1 solubility tag, a 10×Asp spacer sequence, and a tobacco etch virus (TEV) protease cleavage site in cloning site 1. The nsp8 gene was subsequently cloned into cloning site 2 without any tag. The gene for nsp12 was cloned into the pAceBac vector with cleavable 6×His on the C-terminus. The nsp7/nsp8 protein complex was expressed and purified as previously the truncated nsp7/nsp8 in E. coli [Konkolova 2020]. The SARS-CoV-2 nsp12 plasmid was used to prepare recombinant baculovirus Sf9 insect cells were infected at 1-2 × 10^6 cell/ml with the tertiary recombinant baculovirus. After 68 h the cells were collected by centrifugation, resuspended in the lysis buffer (50 mM HEPES 7.4, 300 mM NaCl, 20 mM imidazole, 3 mM $MgCl^2$, 10% (v/v) glycerol, and 3 mM β-mercaptoethanol) and sonicated (Q700 Sonicator, QSonica). The lysate was subsequently cleared by centrifugation and the supernatant was incubated with the Ni-NTA agarose (Thermo Scientific), washed with lysis buffer and finally, the protein was eluted with lysis buffer supplemented with 300 mM imidazole. Nsp12 protein was further purified by size exclusion chromatography using Superdex 200 16/600 (GE Healthcare) in size-exclusion buffer (20 mM HEPES 7.4, 300 mM NaCl, 1 mM $MgCl_2$, 10% (v/v) glycerol and 3 mM β-mercaptoethanol). Fractions containing the pure nsp12 protein were concentrated to 5 mg/ml, flash-frozen, and stored at -80 °C until needed.

Fluorescence-based primer extension polymerase assay

The polymerase activity was determined in a primer extension reaction using a fluorescently labeled primer (HEX-5'-AGAACCUGUUGAACAAAAGC-3') and an RNA template (5'-AUUAUUAGCUGCUUUUGUUCAACAGGUUCU-3'). The polymerase activity assay was performed in the total volume of 10 µl containing the reaction buffer (10 mM Tris pH 8.0, 2 mM $MgCl_2$, 10 mM KCl, 1 mM β-mercaptoethanol), 10 µM NTPs, 0.5 µM T/P complex, 1 µM nsp12 polymerase and 3 µM nsp7/nsp8 complex. The reactions were incubated for 1 h with various concentrations of the inhibitors tested at 30°C and stopped by adding 20 µl of the stop buffer (80% formamide, 50 mM EDTA), samples were denatured at 95 °C for 10 min and primer extension products were separated on a 20 % denaturing polyacrylamide gel (8 M urea, 1× TBE, 20 % acrylamide (19:1) and scanned on the Typhoon 5 Biomolecular Imager (GE Healthcare).

Radioactivity-based primer extension polymerase assay

Was performed as above except the reaction mixture contained0.5 µM T/P complex (P - 5'-AGAACCUGUUGAACAAAAGC-3', T - 5'-U25-GCUUUUGUUCAACAGGUUCU-3') and 0.01µCi/µl [α-32P]-ATP. After incubation 5 µl of the reaction, mixtures were spotted on an anion exchange cellulose filter paper (WhatmanTM Grade DE81 DEAE cellulose paper; GE Healthcare) in triplicates. The Whatman filter was then dried, subsequently washed by 0.125 mM Na_2HPO_4, water, and ethanol, and dried again. Dry filter paper was then analysed using phosphorimaging, the plate was scanned on Amersham Typhoon 5 Biomolecular Imager (GE Healthcare), products were quantified with Image Studio Lite (LI-COR), and the data were processed using GraphPad version 6 (GraphPad Prism version 6, GraphPad Software, San Diego, CA).

Viruses and cell lines

Two representatives of the *Coronaviridae* family, i.e., SARS-CoV-2 (strain SARS-CoV-2/human/Czech Republic/951/2020 isolated from a clinical sample at the National Institute of Health, Prague, Czech Republic, and kindly provided by Dr. Jan Weber, Institute of Organic Chemistry and Biochemistry, Prague, Czech Republic) and feline infectious peritonitis virus (FIPV, ATCC VR990, a pathogen of domestic cats and other felines) were used for our antiviral cell-based studies.

Vero cells (ATCC CCL-81, African Green Monkey, adult kidney, epithelial) and Vero E6 cells (ATCC CRL-1586) were cultured in Dulbecco′s modified Eagle′s medium (DMEM) supplemented with 10% newborn calf serum, 100 U/mL penicillin, 100 µg/mL streptomycin, and 1% glutamine (Sigma-Aldrich, Prague, Czech

Republic) at 37°C and 5% CO_2. Colorectal adenocarcinoma cells (CaCo-2, ATCC HTB-37) were grown in DMEM medium, containing, 20 % newborn calf serum with 100 U/mL penicillin, 100 µg/mL streptomycin, and 1 % L-glutamine (Sigma-Aldrich, Prague, Czech Republic) at 37°C and 5% CO_2. Felis catus kidney cortex cells (CRFK, ATCC CCL-94) were grown in DMEM supplemented with 10% newborn calf serum, 100 U/mL penicillin, 100 µg/mL streptomycin, and 1% glutamine (Sigma-Aldrich, Prague, Czech Republic) at 37°C and 5% CO_2. Vero (ATCC CCL-81), CaCo-2 (ATCC HTB-37), and CRFK (ATCC CCL-94) cells were used for antiviral and cytotoxicity assays, and Vero E6 cells (ATCC CRL-1586) were used to perform plaque assays.

Cytotoxicity studies

Vero (ATCC CCL-81), CaCo-2 (ATCC HTB-37), and CRFK (ATCC CCL-94) cells were seeded into each well of the 96-well microtitrate plate (approx. 2×10^4 cells per a well) and were incubated for 24 hours at 37 °C and 5% CO_2. Cell monolayers in 96-well plates were treated with compounds HeE1-2Tyr (16), 17, or 18 at the concentration of 50 µM (for the initial screening) or with compounds HeE1-2Tyr (16) and 17 in concentration ranges from 0 to 50 µM (for dose-response studies; 2-fold dilutions, three wells per concentration) and cultured for 48 hours. Cells treated with remdesivir (at the same concentrations) or DMSO (1% w/w) were used as positive and negative controls, respectively. The cytotoxic activity of the compounds was determined in terms of cell viability using the Cell Counting Kit-8 (Dojindo Molecular Technologies, Munich, Germany) following the Manufacturer's instructions. The assay is based on quantitative reduction of WST-8 tetrazolium salt to yellow formazan by cellular dehydrogenases. Cell viability was estimated as the percentage of colorimetric absorbance at 450 nm by the compound-treated cells relative to the absorbance by mock-treated cells. The concentration of compound that reduced cell viability by 50% was considered the 50% cytotoxic concentration (CC_{50}).

Antiviral efficacy of the studied compounds in cell-based assays

To study the antiviral effects of compounds HeE1-2Tyr (16), 17, and 18, we used a viral titer reduction assay. Vero (ATCC CCL-81), CaCo-2 (ATCC HTB-37), and CRFK (ATCC CCL-94) cells were seeded into each well of the 96-well microtitrate plate (approx. 2×10^4 cells per a well) and were incubated for 24 hours at 37 °C and 5% CO_2. Then, the medium was aspirated, replaced with 200 µl of fresh medium containing compounds HeE1-2Tyr (16), 17, or 18 at the concentration of 50 µM (for the initial screening) or with compounds HeE1-2Tyr (16) or 17 in the concentration range of 0 to 50 µM (for dose-response antiviral studies; 2-fold dilution, three wells per compound). The treated cells were simultaneously inoculated with SARS-CoV-2 (for Vero and CaCo-2 cells) or FIPV (for CRFK cells) at an MOI of 0.1 and incubated for an additional 48 hours. Virus-infected cells treated with remdesivir (at the same concentrations) or DMSO (1% w/w) were used as positive and negative controls, respectively. Viral titers were determined from the collected supernatant media by a plaque assay and used to construct dose-response curves and for calculation of the 50% effective concentrations (EC_{50}; the concentration of compound required to inhibit the viral titer by 50% compared to the control value).

Plaque assay

Plaque assays were performed using Vero E6 cells (ATCC CRL-1586) as described previously (De Madrid and Porterfield, 1969; Eyer et al., 2015). Briefly, 10-fold dilutions of SARS-CoV-2 or FIPV were prepared in 24-well tissue culture plates, and the cells were added to each well (0.6–1.5 × 10^5 cells per well). After a 4-hour incubation at 37 °C and 5% CO_2, the suspension was overlaid with 1.5% (w/v) carboxymethylcellulose in a two-fold concentrated DMEM medium. Following a 5-day incubation at 37 °C and 5% CO_2, the infected plates were washed with phosphate-buffered saline, and the cell monolayers were stained with naphthalene black. The virus titer was expressed as plaque-forming units (PFU)/ml.

References

1. B. V. L. Potter, L. W. L. Woo, A. Purohit, M. J. Reed, **2003**, WO2003045925.
2. G. Manfroni, F. Meschini, M. L. Barreca, P. Leyssen, A. Samuele, N. Iraci, S. Sabatini, S. Massari, G. Maga, J. Neyts, V. Cecchetti (2012). *Bioorg. Med. Chem*. **2012**, *20*(2), 866-876.
3. D. Tarantino, R. Cannalire, E. Mastrangelo, R. Croci, G. Querat, M. L. Barreca, M. Bolognesi, G. Manfroni, V. Cecchetti, M. Milani, *Antiviral Research* **2016**, *134*, 226-235.

NMR spectra

5-(benzyloxy)-2-methylbenzo[*d*]thiazole (**2**)

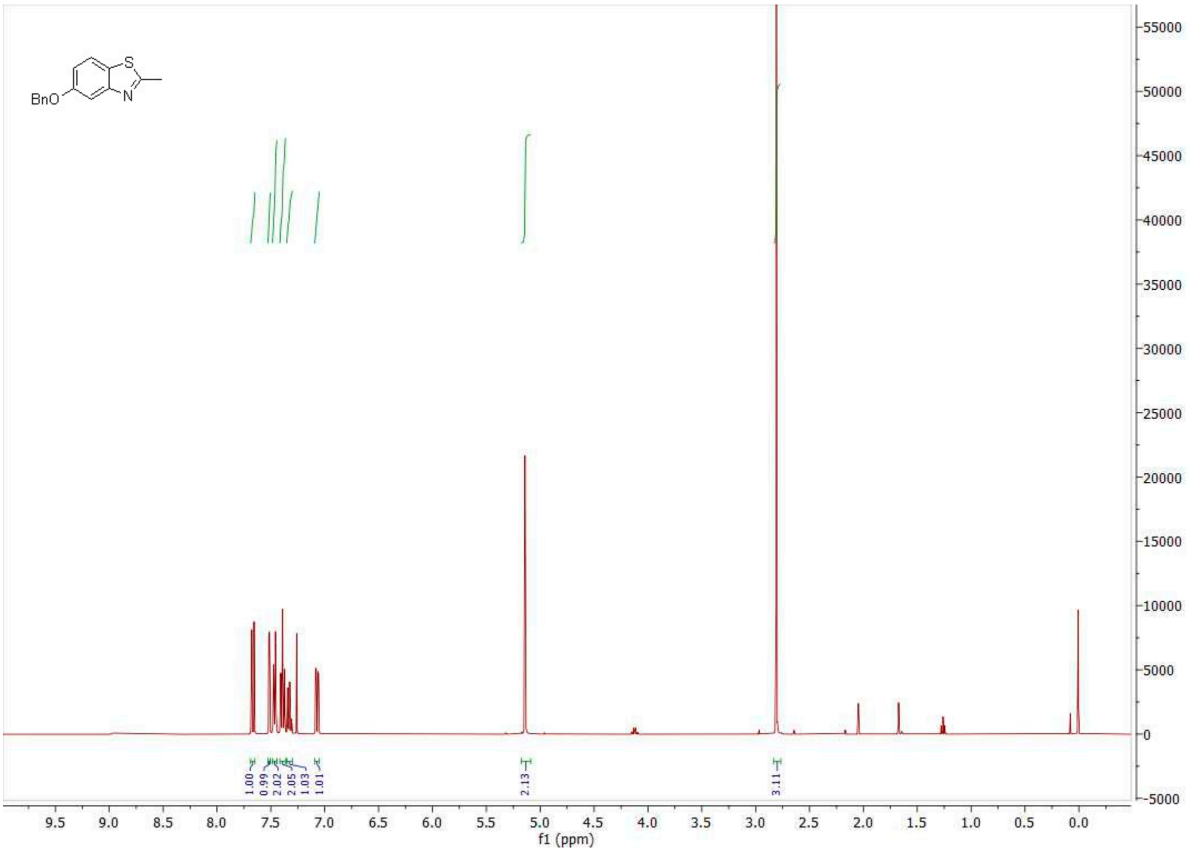

Ethyl 2-(5-(benzyloxy)benzo[*d*]thiazol-2-yl)acetate (**3**)

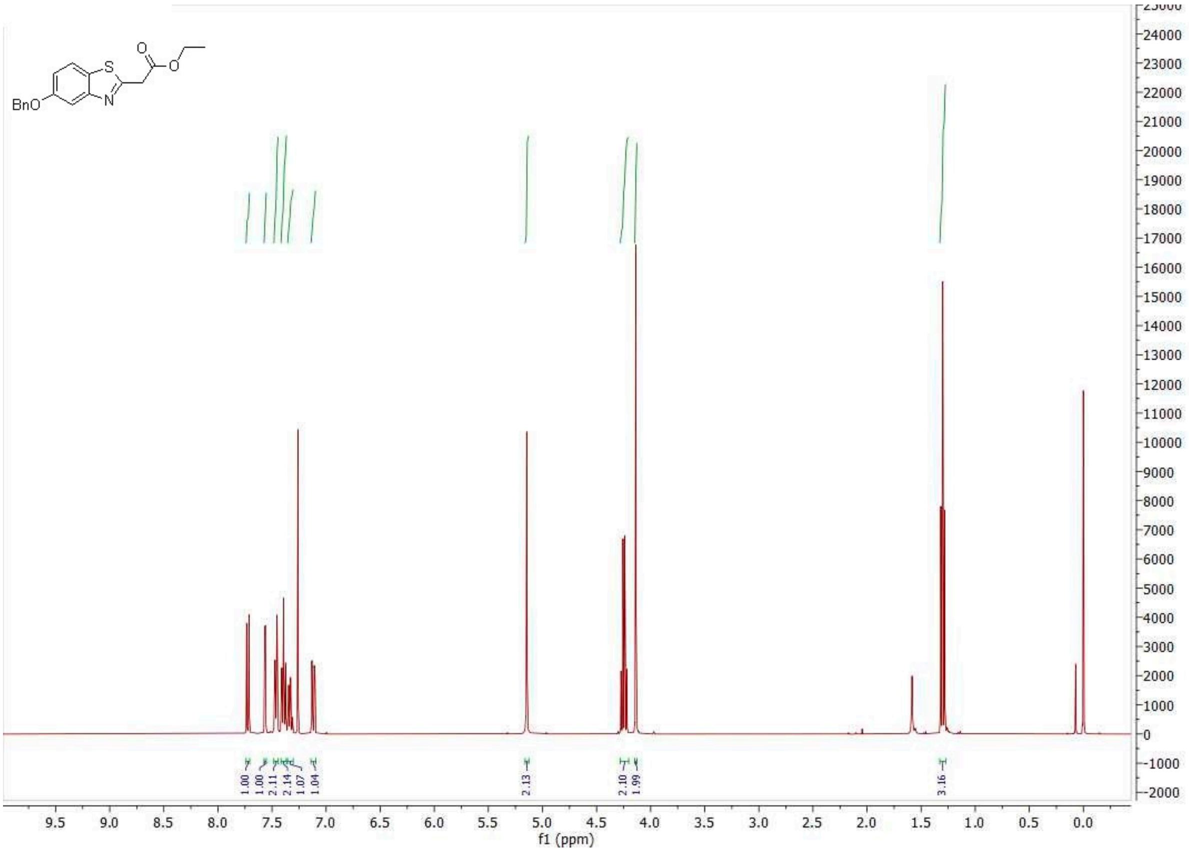

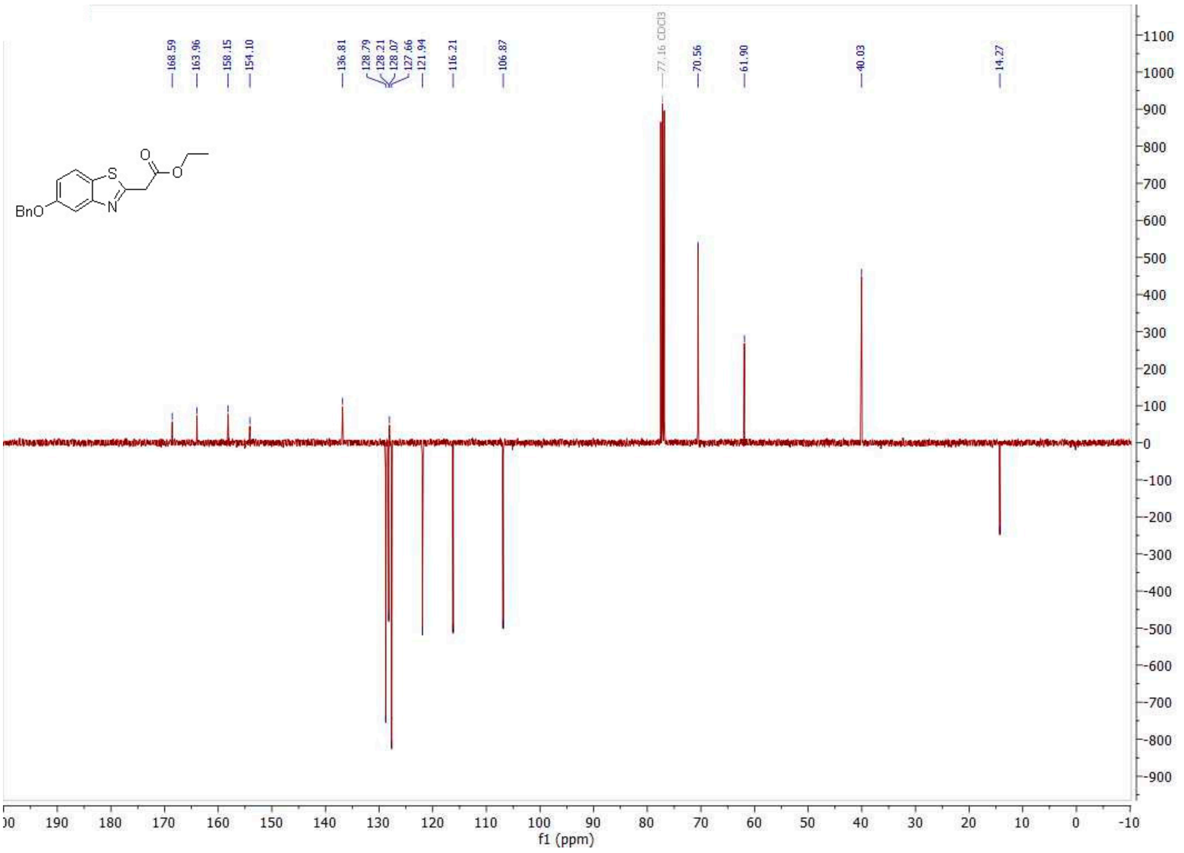

Ethyl 8-(benzyloxy)-1-oxo-2-phenyl-1*H*-benzo[4,5]thiazolo[3,2-a]pyridine-4-carboxylate (**5**)

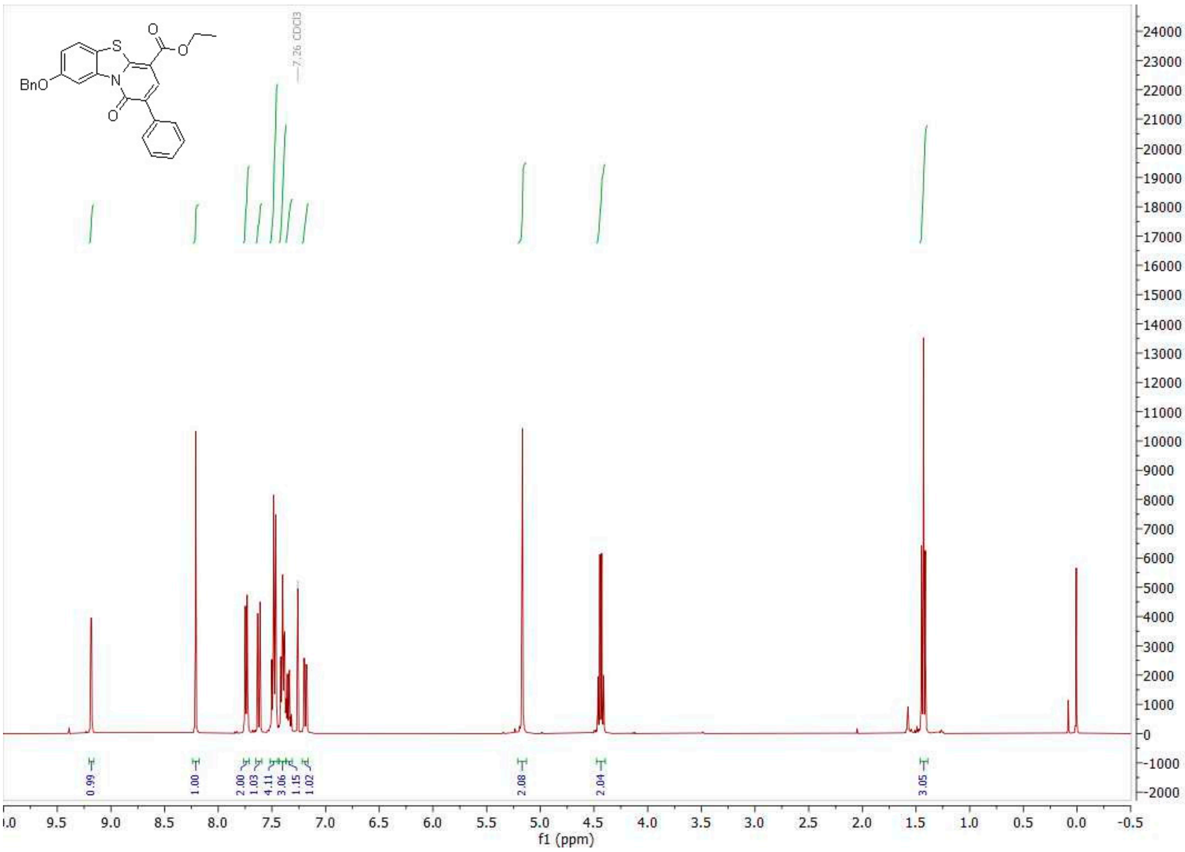

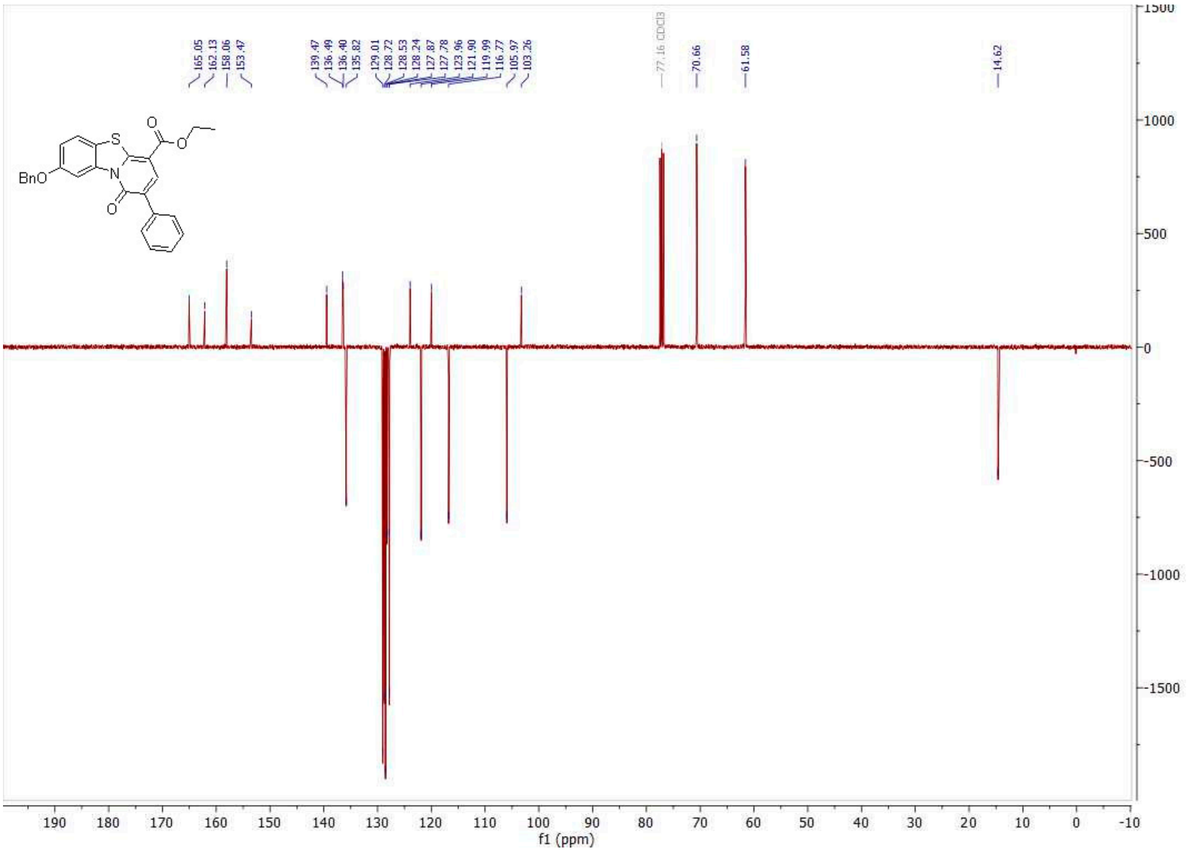

Ethyl 8-hydroxy-1-oxo-2-phenyl-1*H*-benzo[4,5]thiazolo[3,2-a]pyridine-4-carboxylate (**6**)

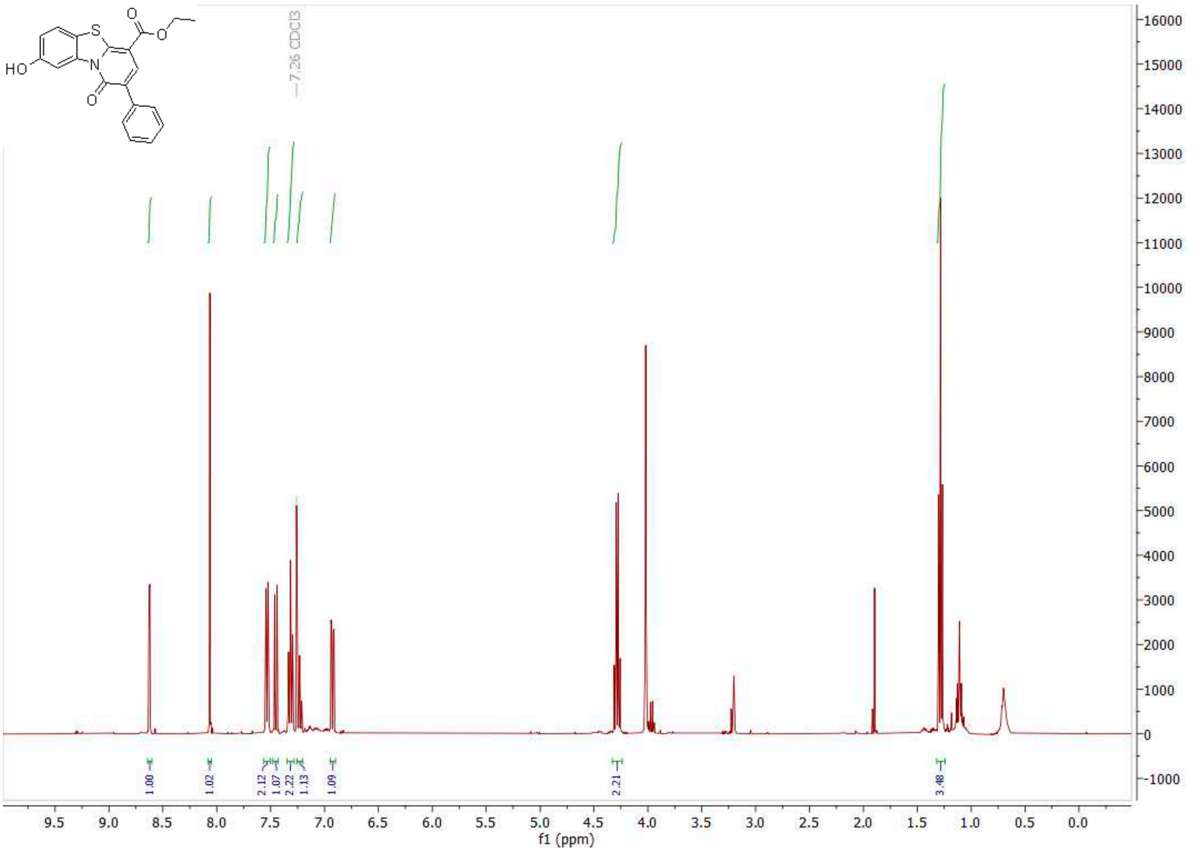

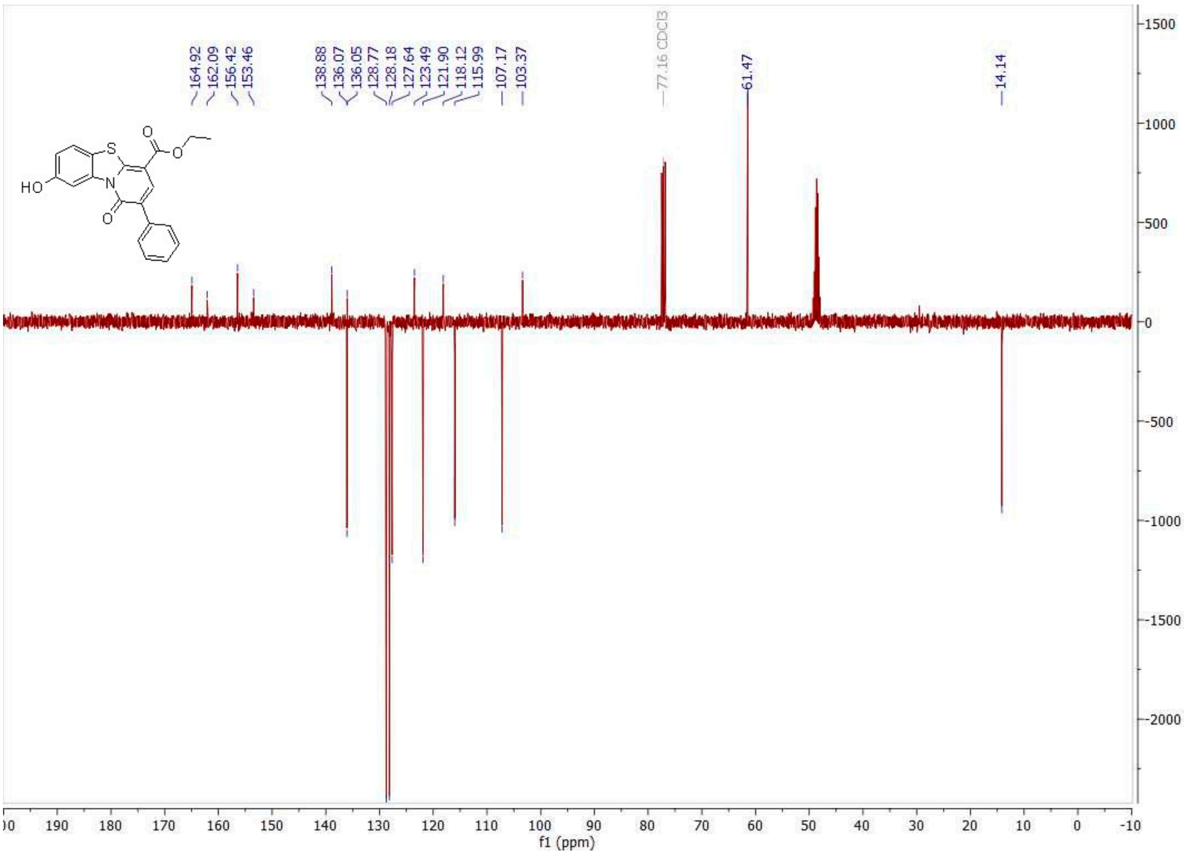

Ethyl 8-(benzyloxy)-1-oxo-1*H*-benzo[4,5]thiazolo[3,2-a]pyridine-4-carboxylate (**7**)

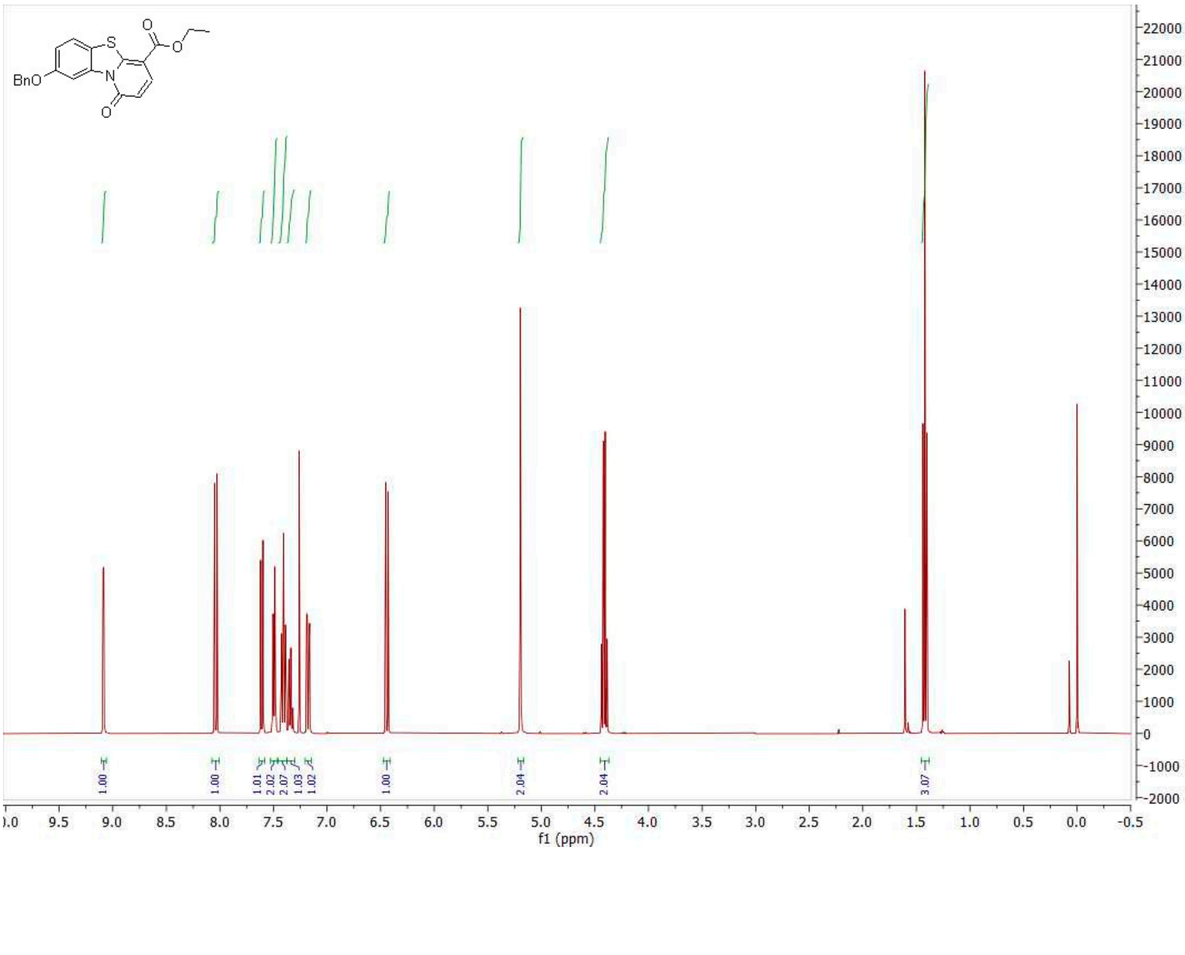

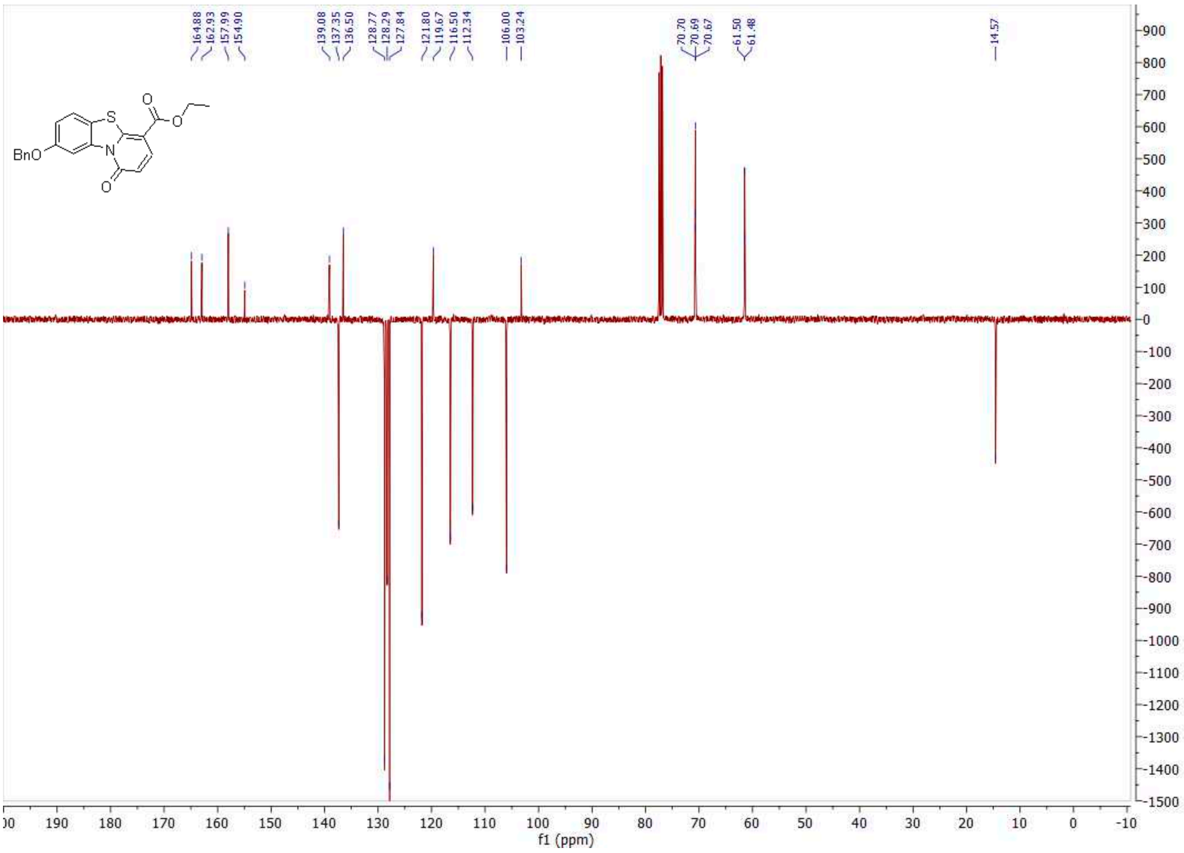

Ethyl 8-hydroxy-1-oxo-1*H*-benzo[4,5]thiazolo[3,2-a]pyridine-4-carboxylate (**8**)

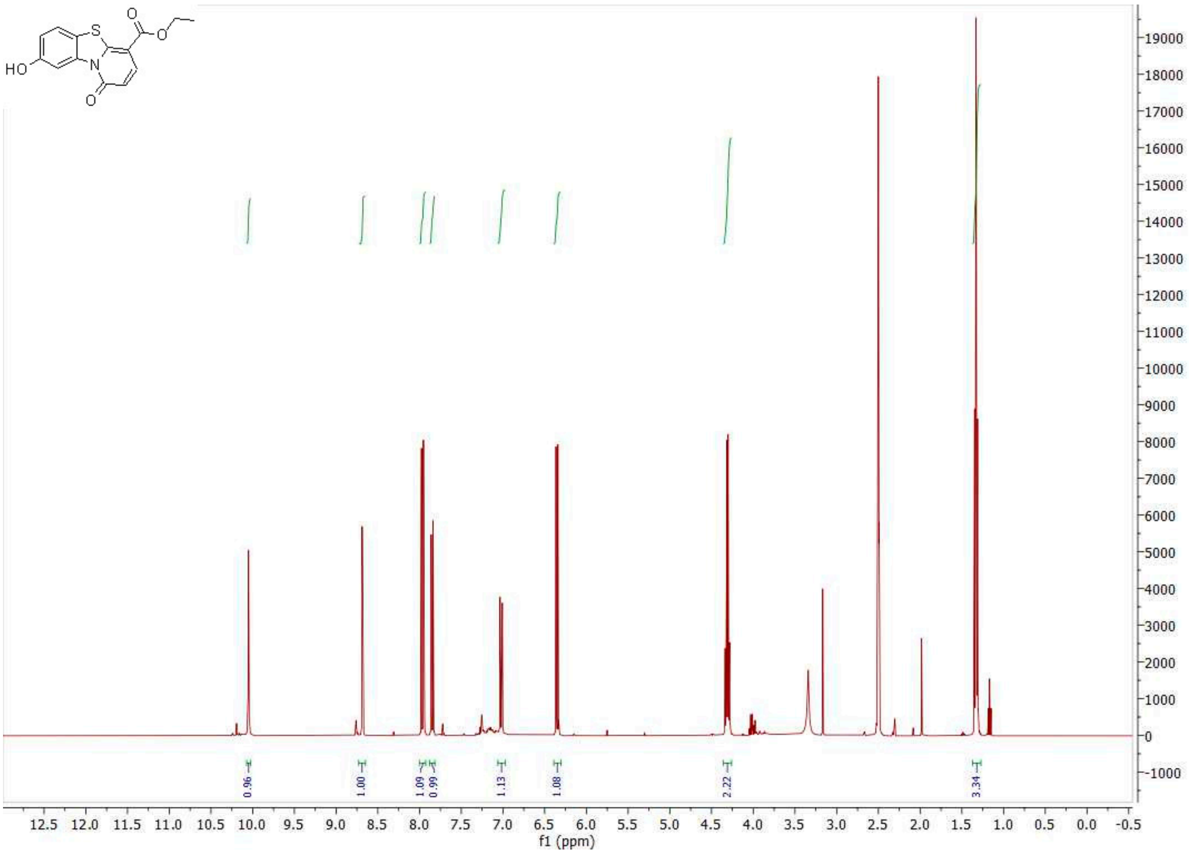

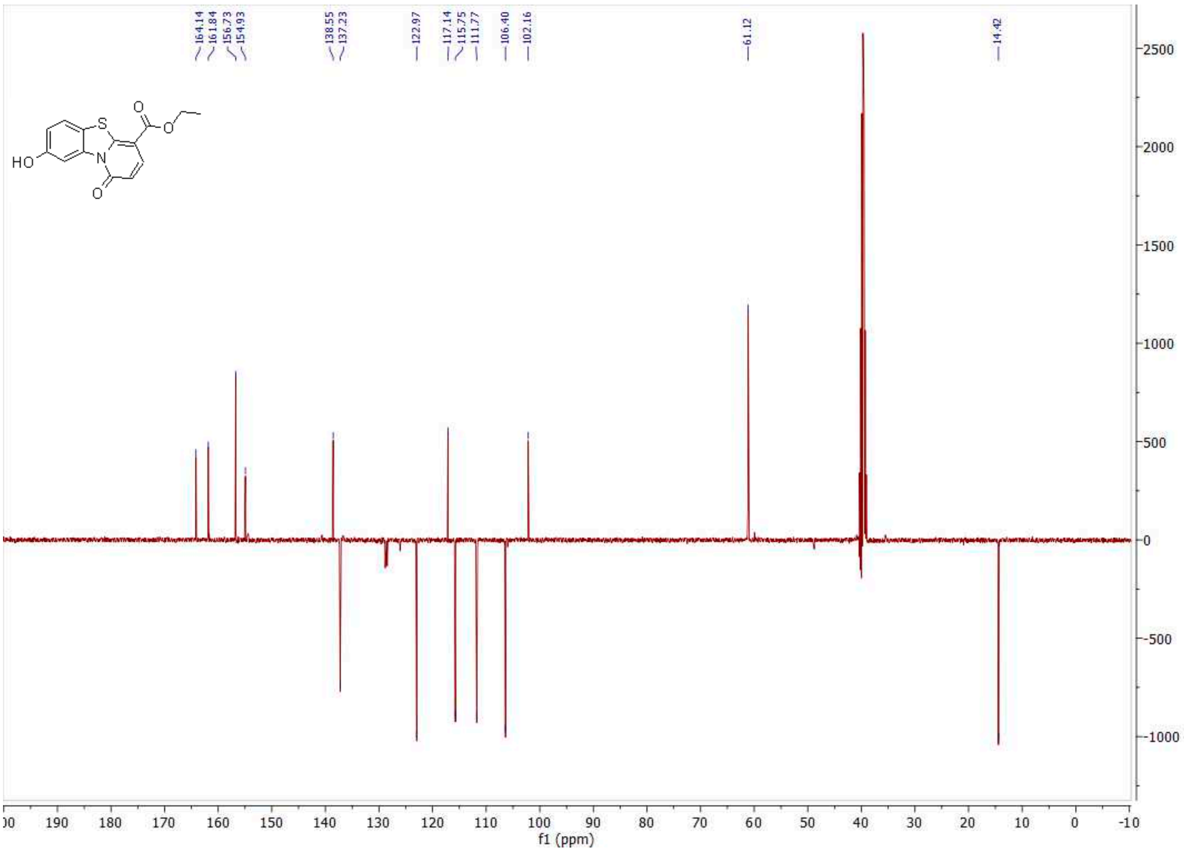

Ethyl 8-(cyclohexyloxy)-1-oxo-2-phenyl-1*H*-benzo[4,5]thiazolo[3,2-a]pyridine-4-carboxylate (**9**)

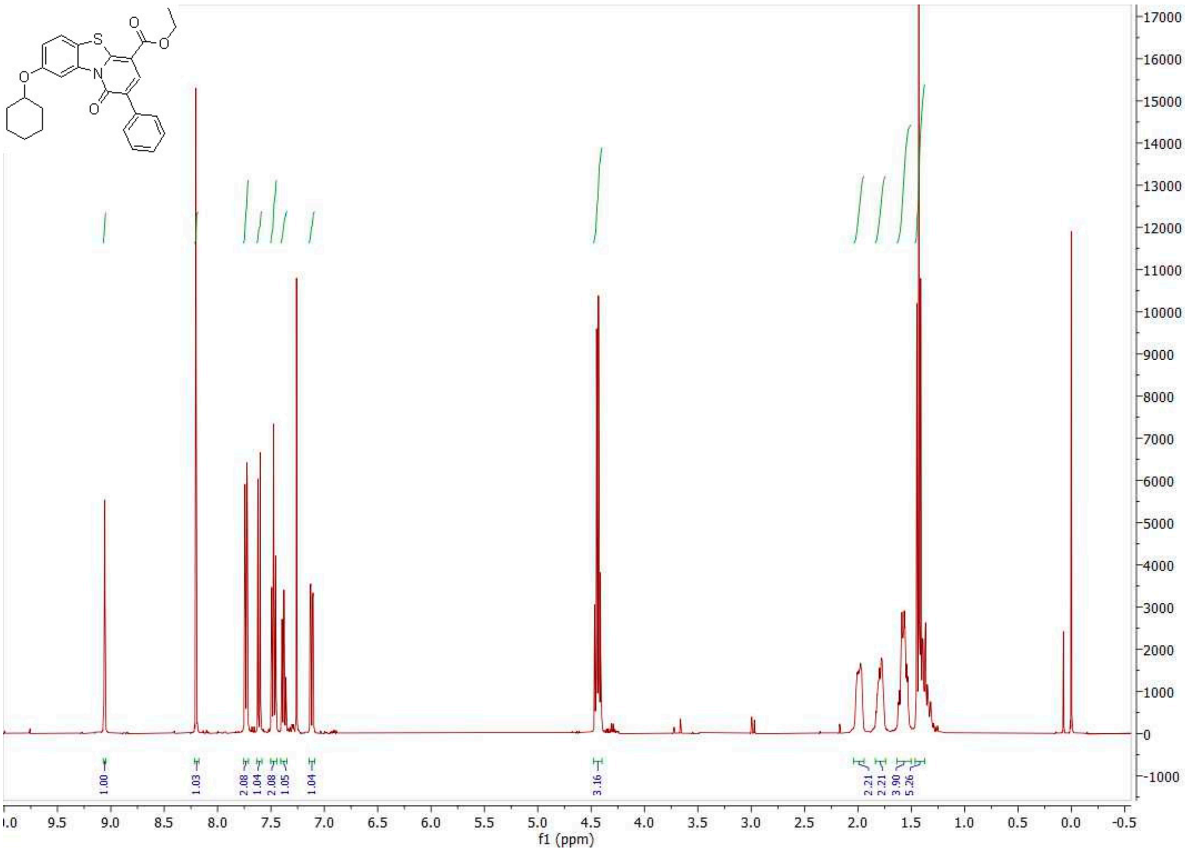

8-(cyclohexyloxy)-1-oxo-2-phenyl-1*H*-benzo[4,5]thiazolo[3,2-a]pyridine-4-carboxylic acid (**10**)

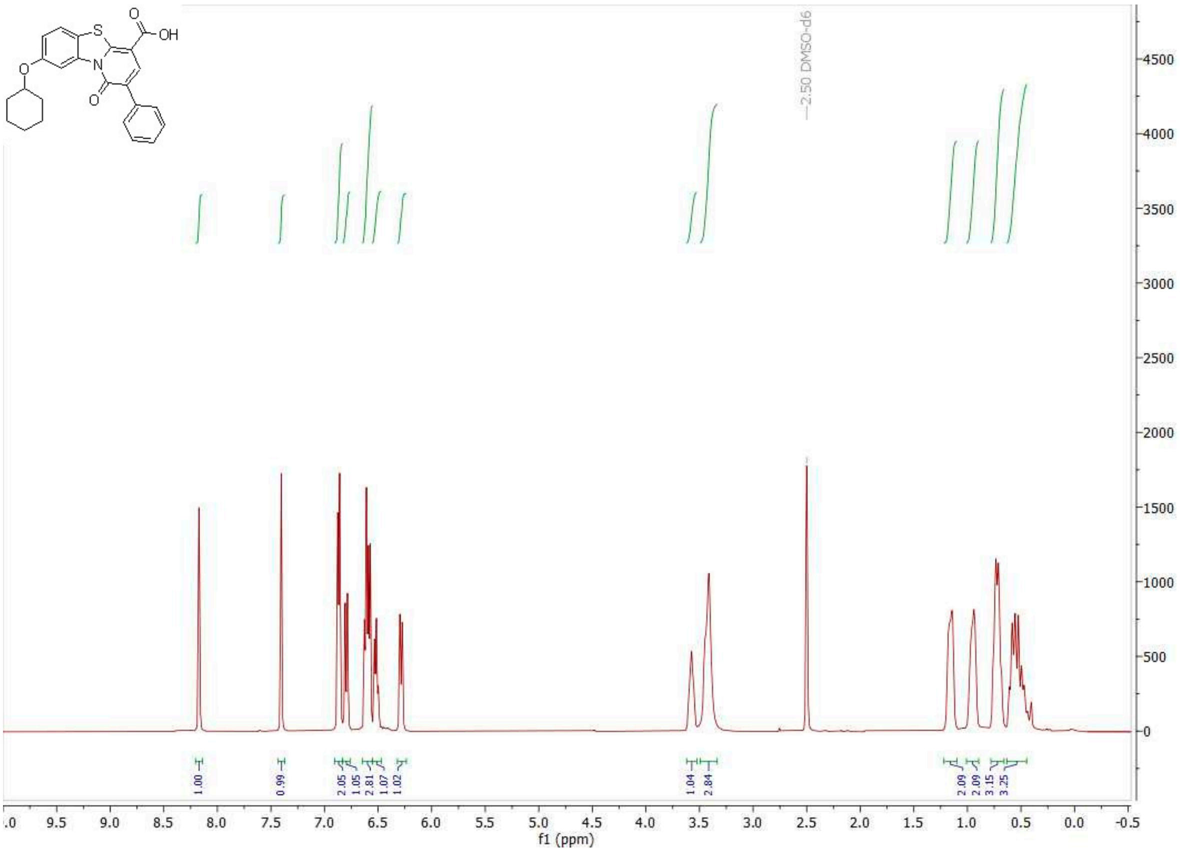

Ethyl 8-(cyclohexyloxy)-1-oxo-1*H*-benzo[4,5]thiazolo[3,2-a]pyridine-4-carboxylate (**11**)

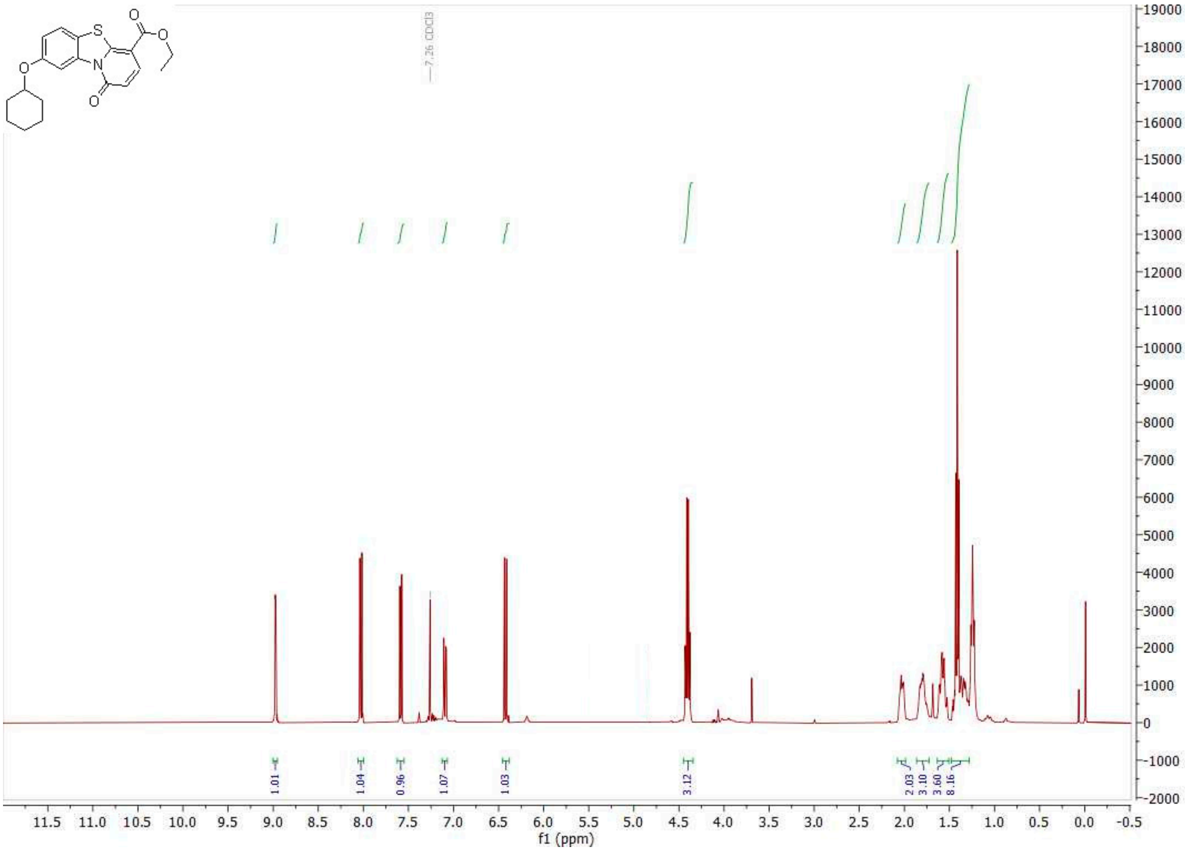

8-(cyclohexyloxy)-1-oxo-1*H*-benzo[4,5]thiazolo[3,2-a]pyridine-4-carboxylic acid (**12**)

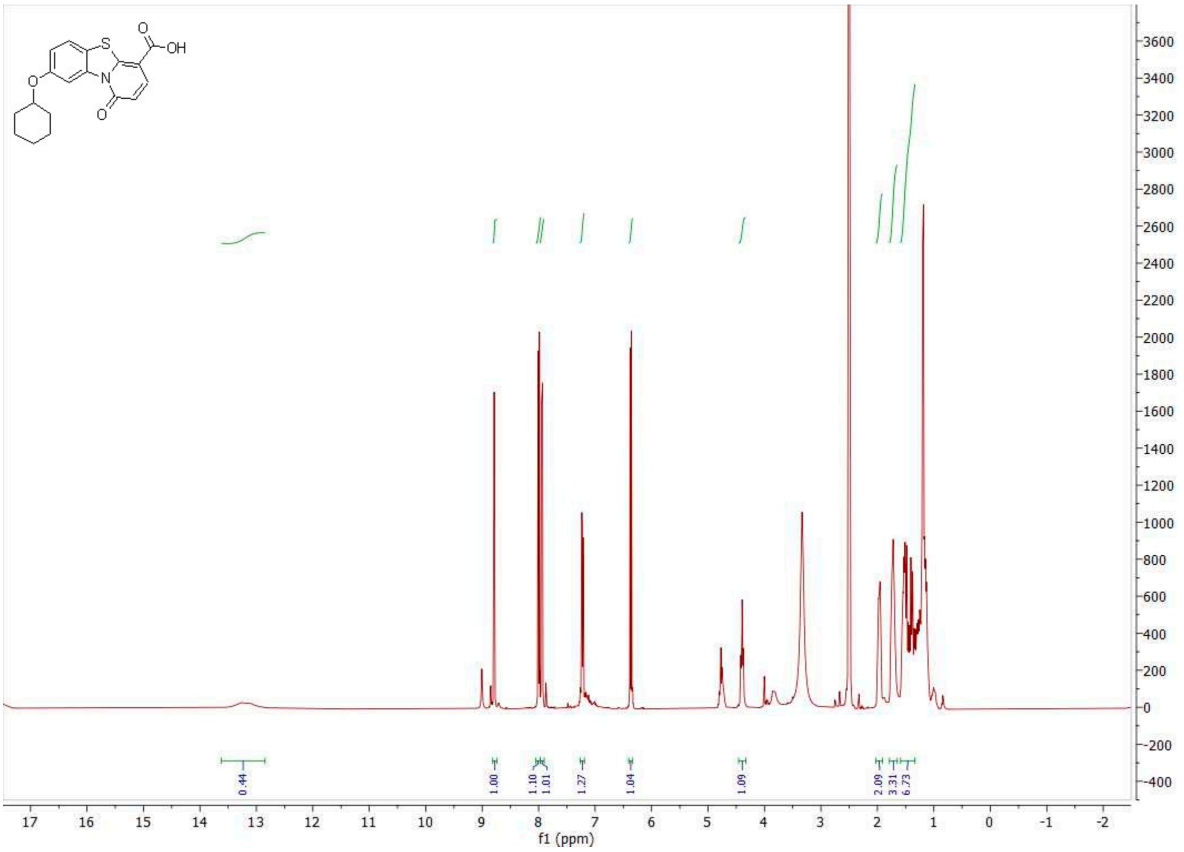

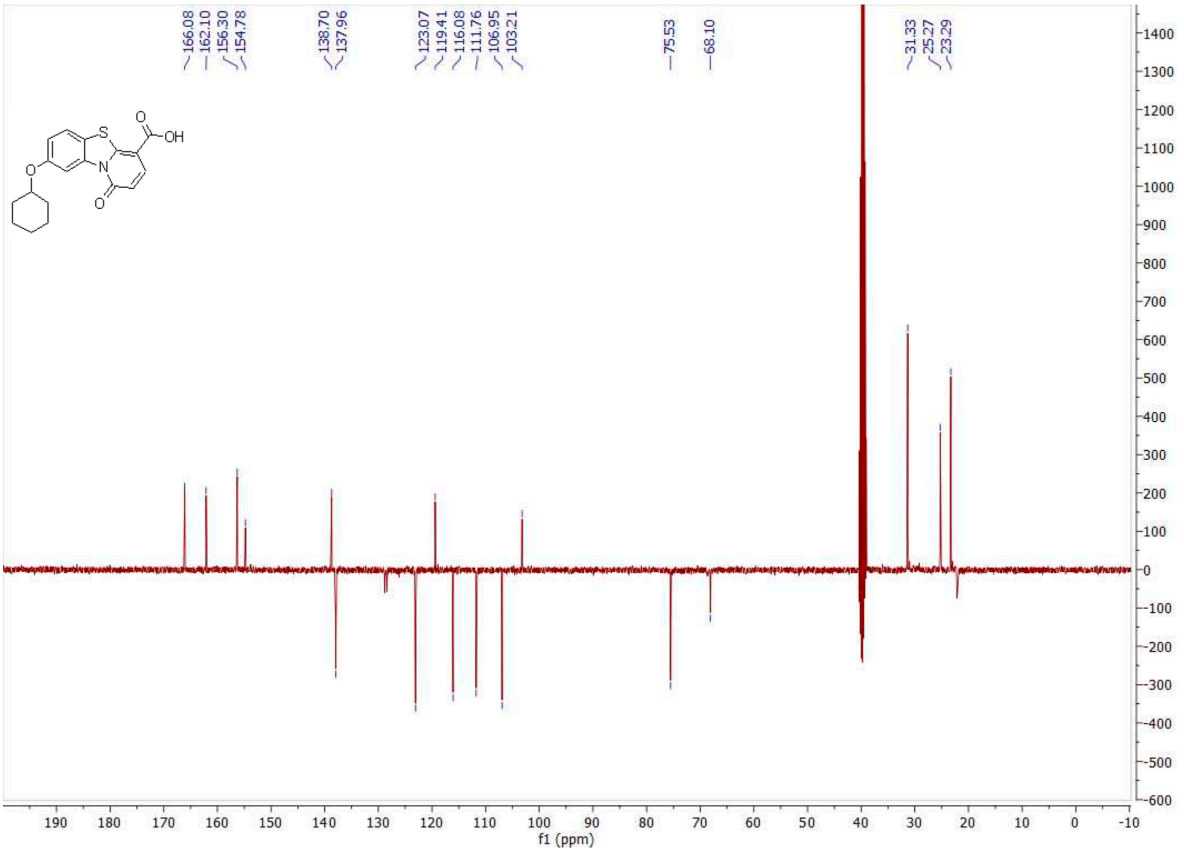

Methyl (8-(cyclohexyloxy)-1-oxo-2-phenyl-1*H*-benzo[4,5]thiazolo[3,2-a]pyridine-4-carbonyl)-*L*-tyrosinate (**13**)

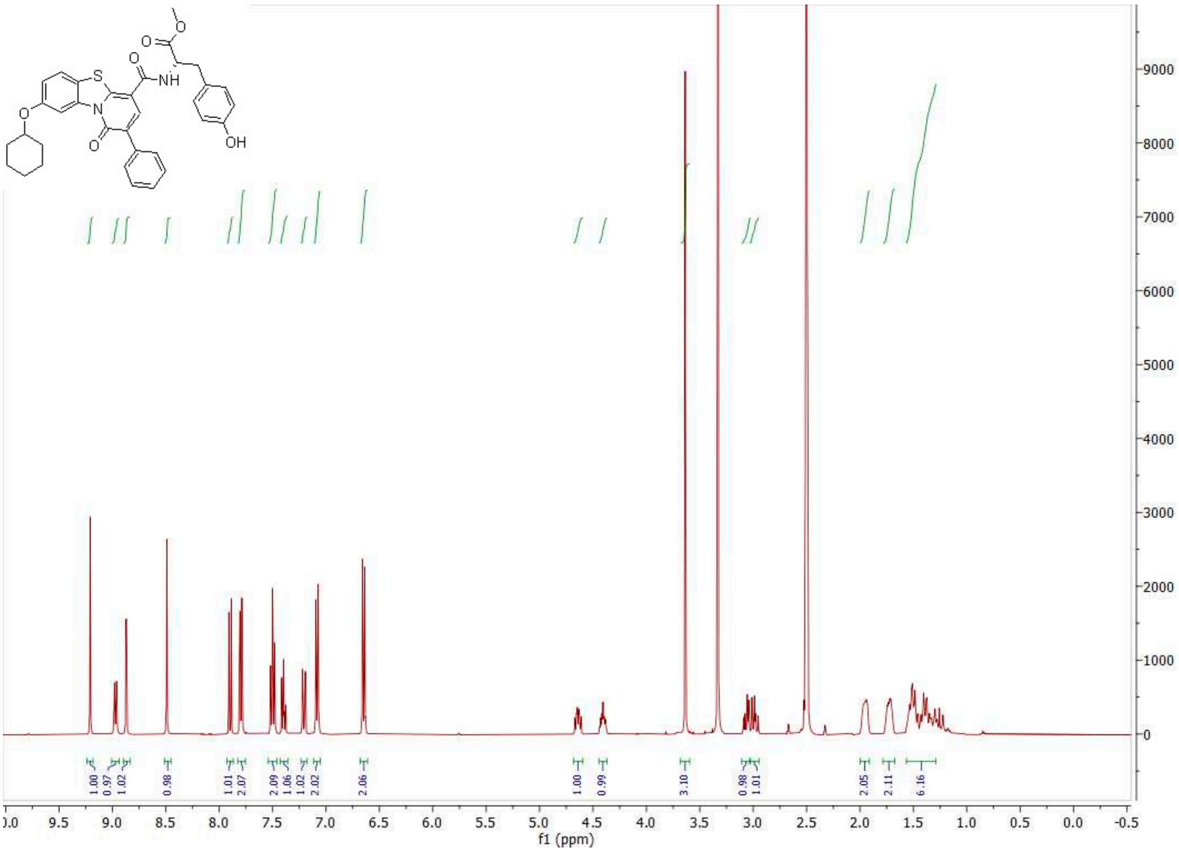

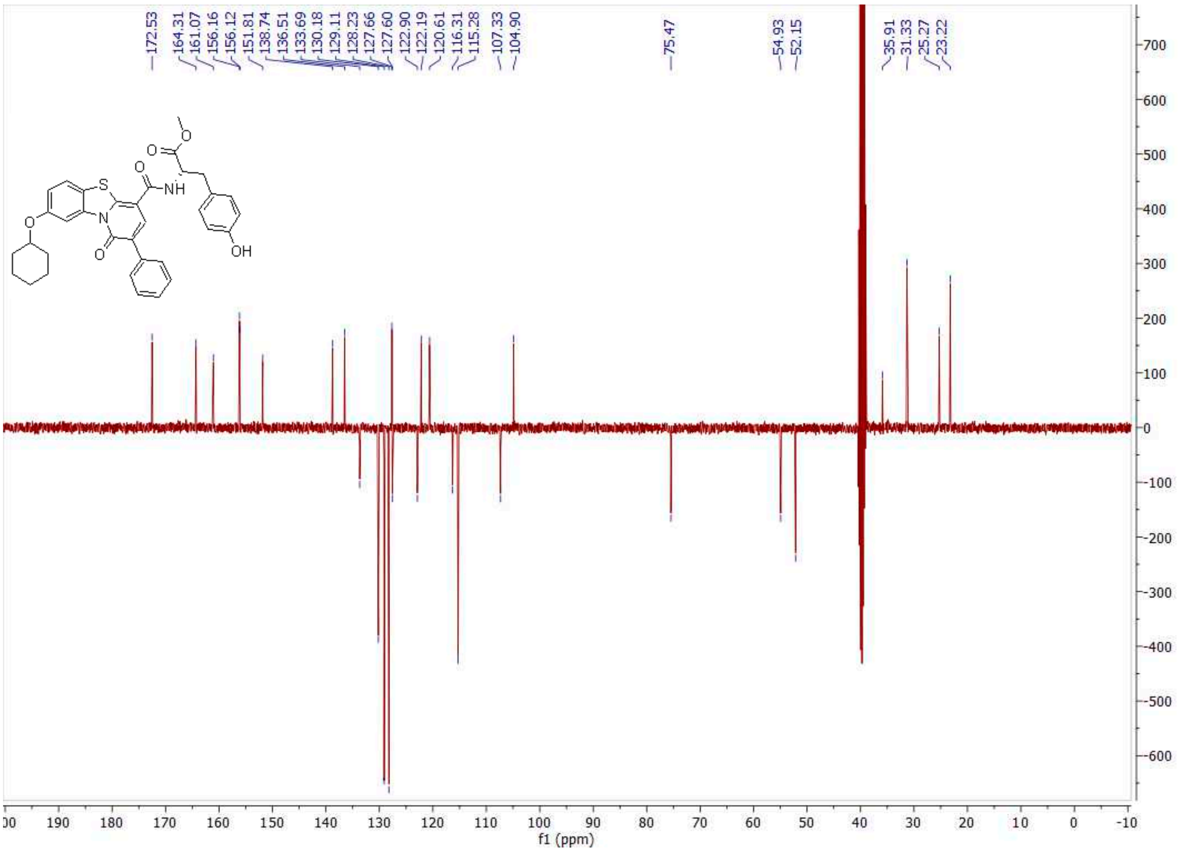

Methyl (8-(cyclohexyloxy)-1-oxo-2-phenyl-1*H*-benzo[4,5]thiazolo[3,2-a]pyridine-4-carbonyl)-*L*-phenylalaninate (**14**)

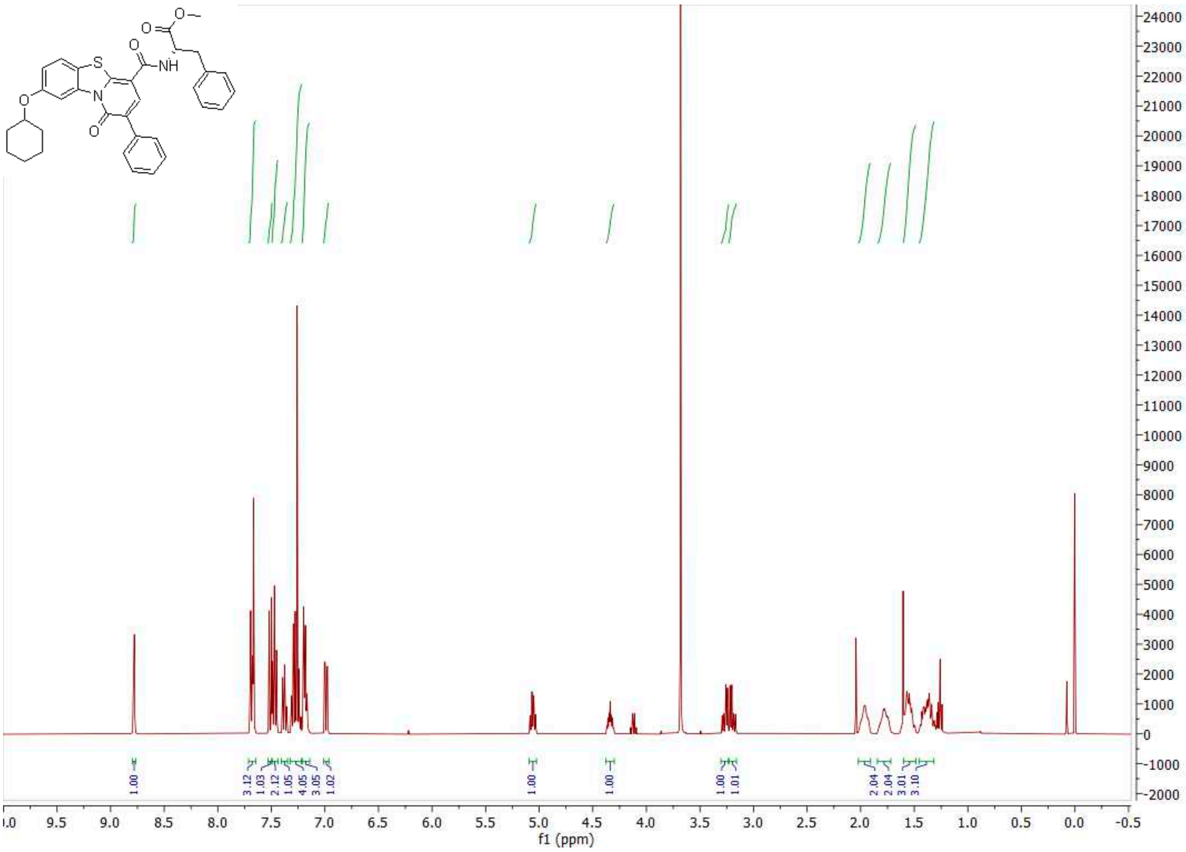

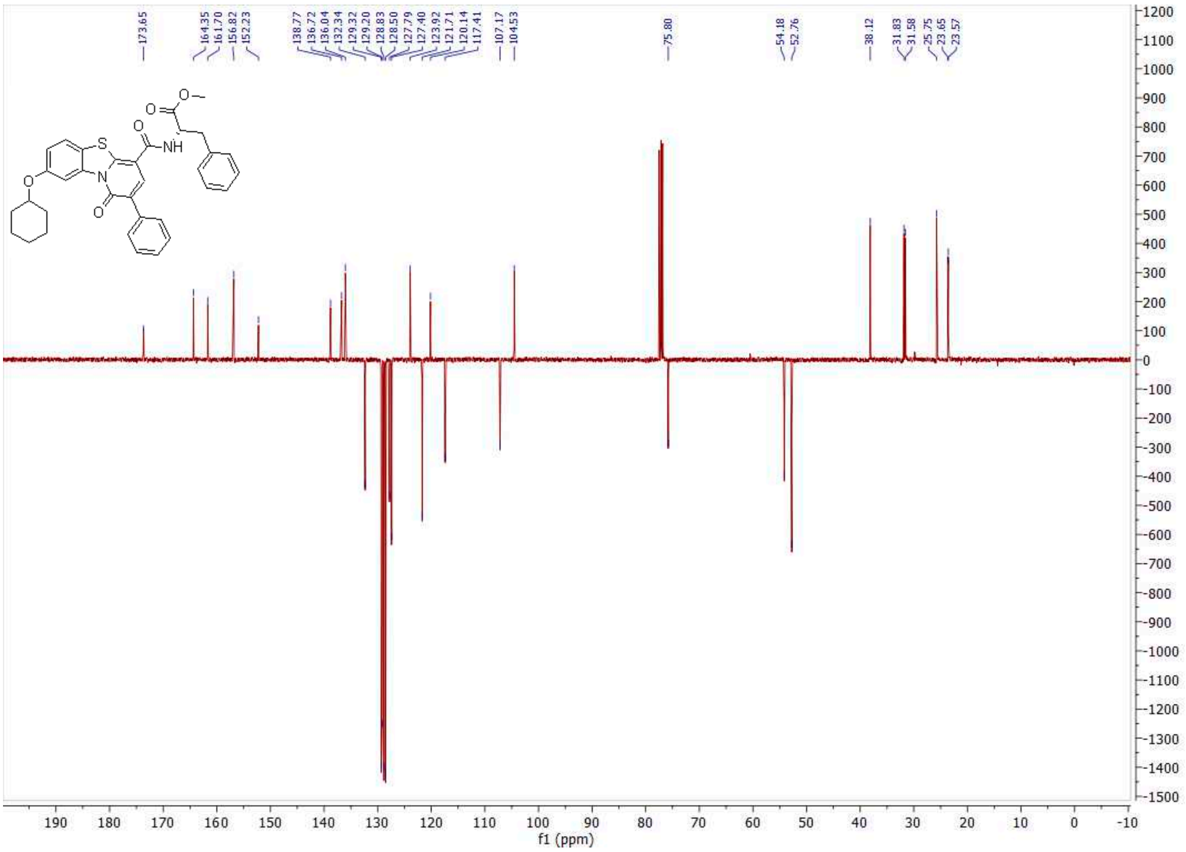

Methyl (8-(cyclohexyloxy)-1-oxo-1*H*-benzo[4,5]thiazolo[3,2-a]pyridine-4-carbonyl)-*L*-tyrosinate (**15**)

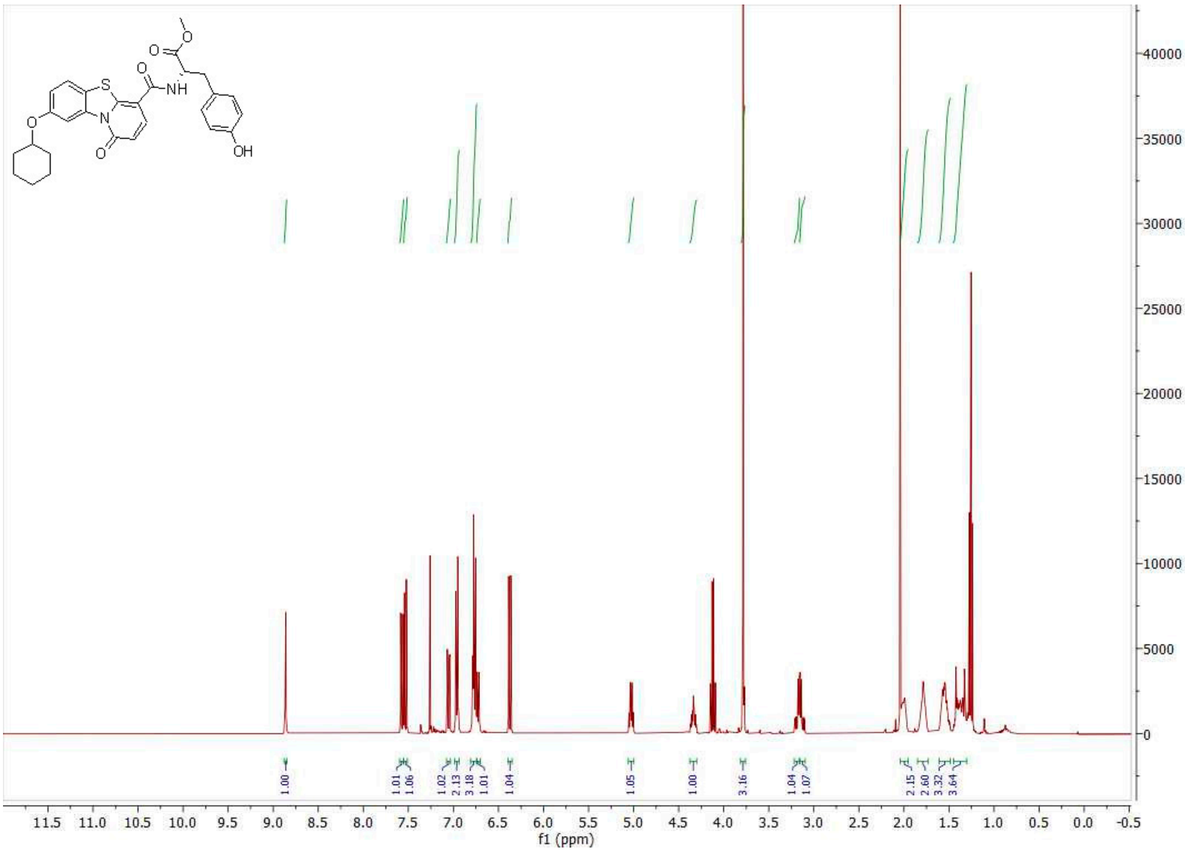

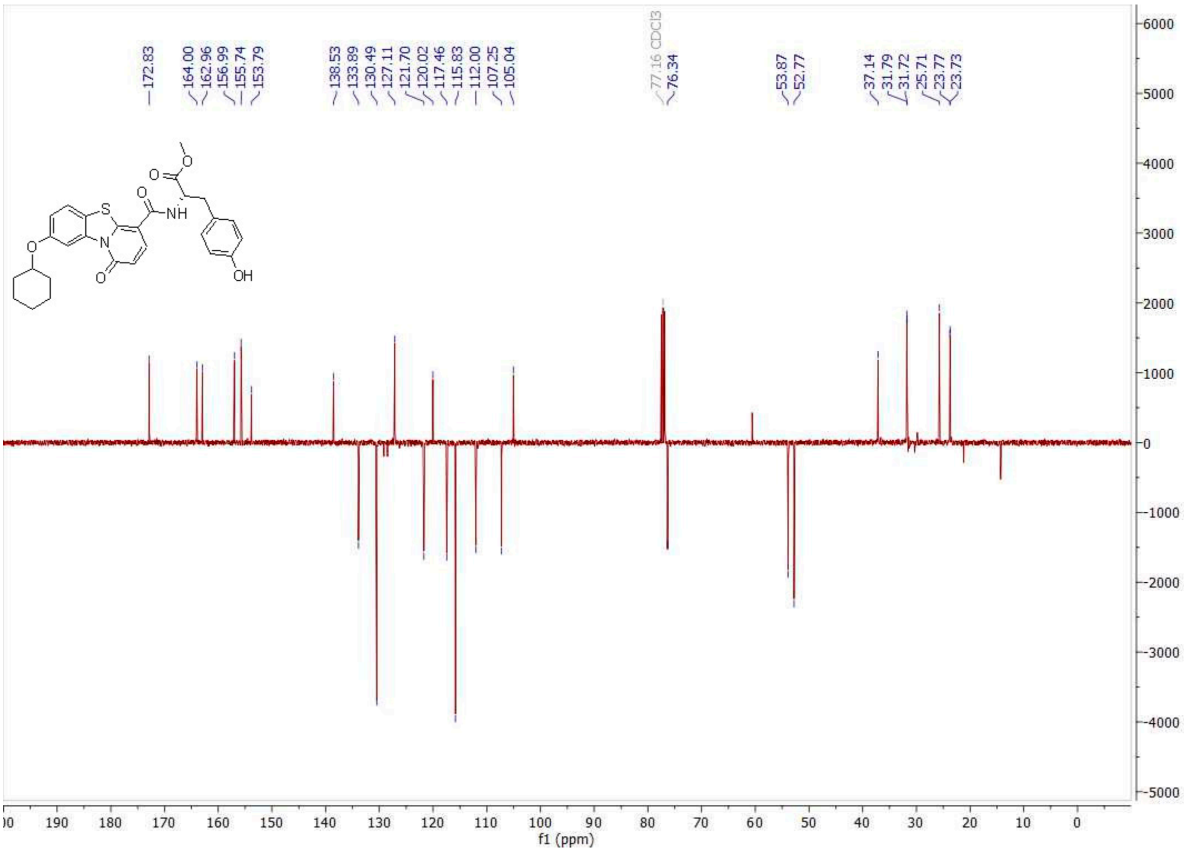

(8-(cyclohexyloxy)-1-oxo-2-phenyl-1*H*-benzo[4,5]thiazolo[3,2-a]pyridine-4-carbonyl)-*L*-tyrosine (**16**)

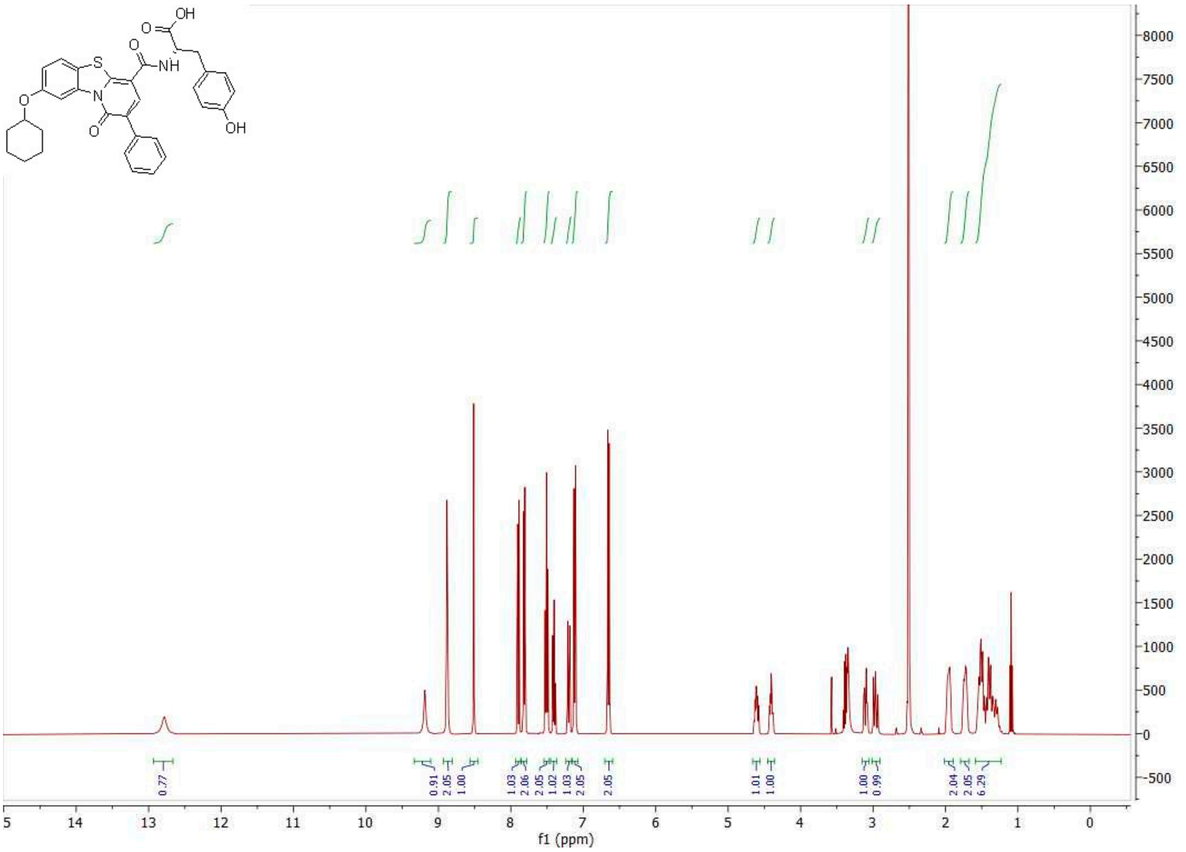

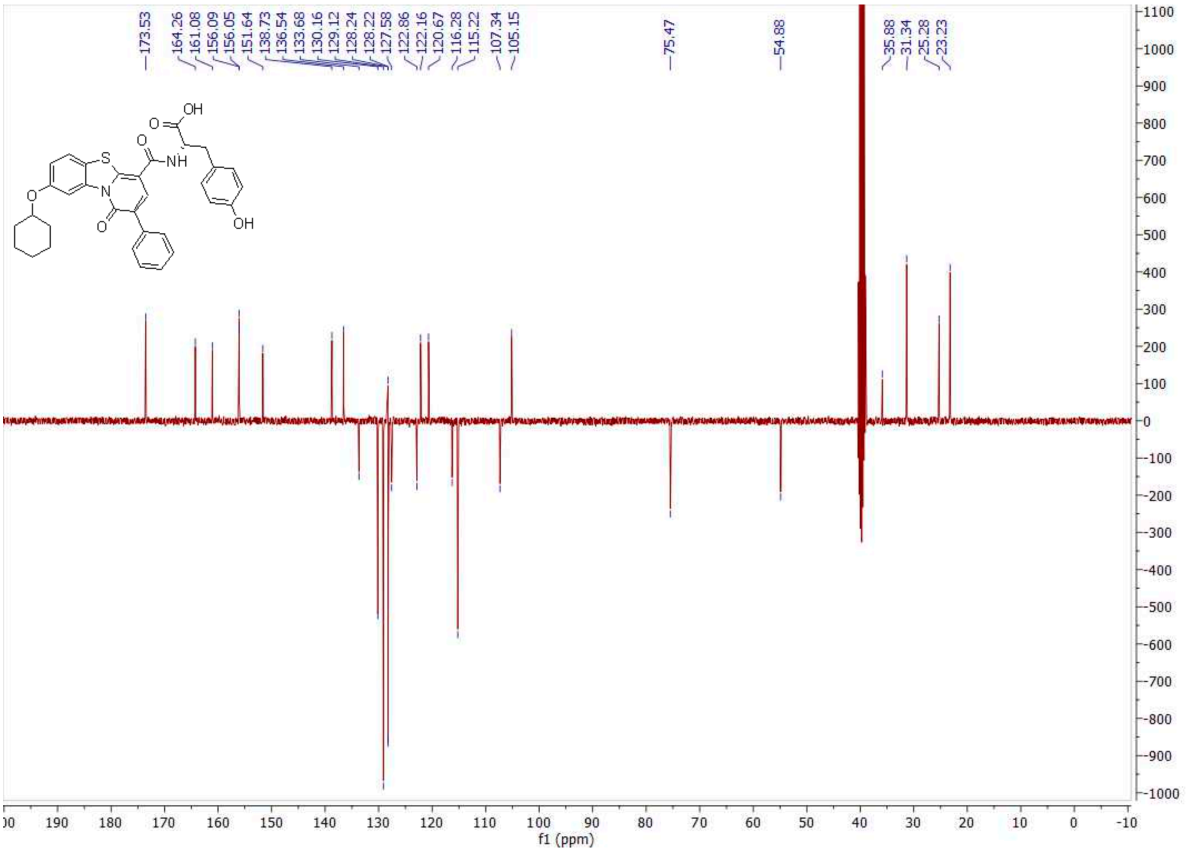

(8-(cyclohexyloxy)-1-oxo-2-phenyl-1*H*-benzo[4,5]thiazolo[3,2-a]pyridine-4-carbonyl)-*L*-phenylalanine (**17**)

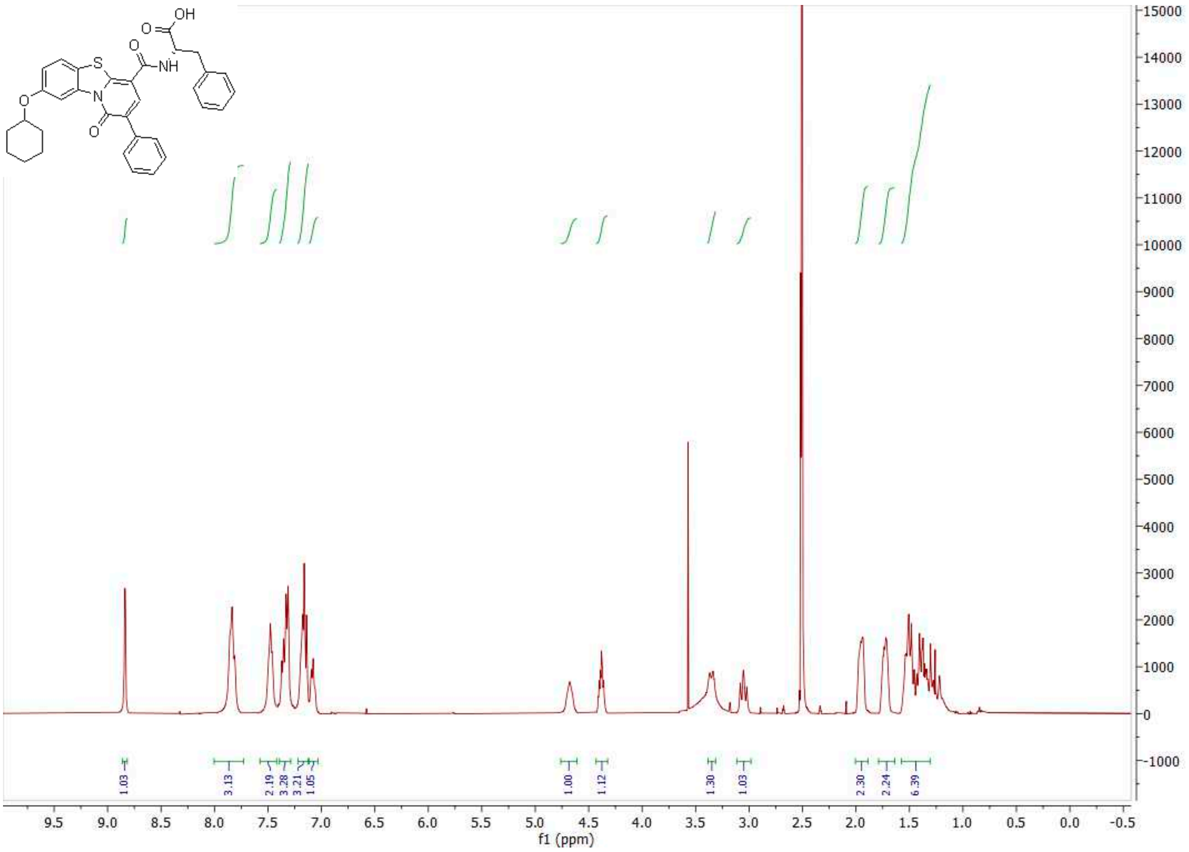

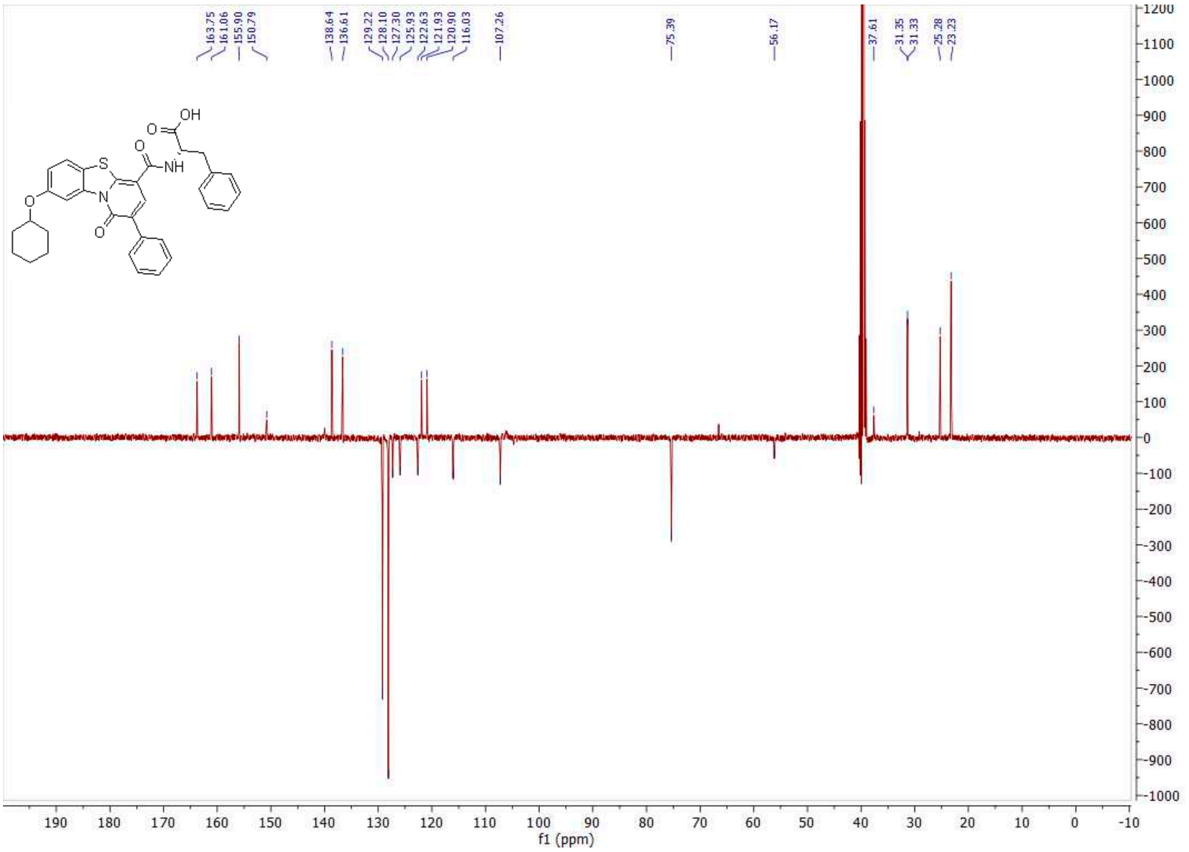

(8-(cyclohexyloxy)-1-oxo-1*H*-benzo[4,5]thiazolo[3,2-a]pyridine-4-carbonyl)-*L*-tyrosine (**18**)

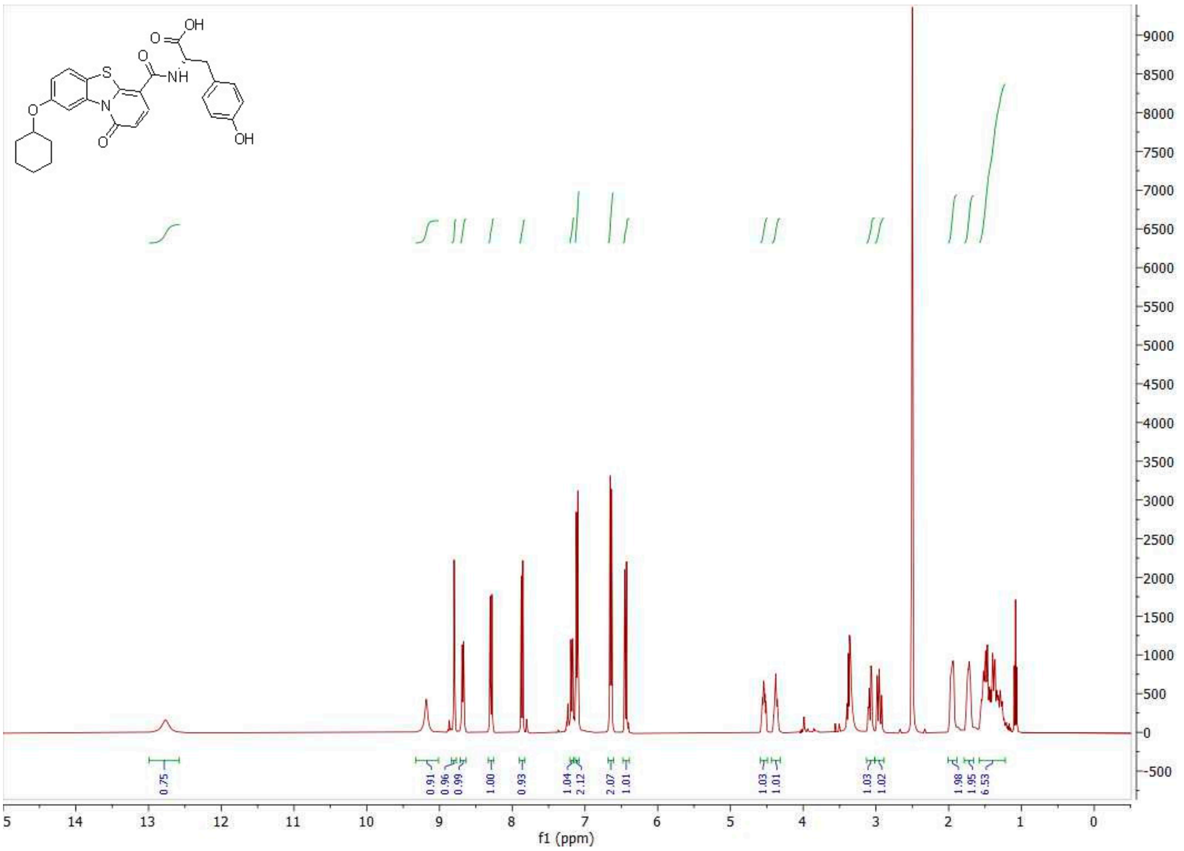

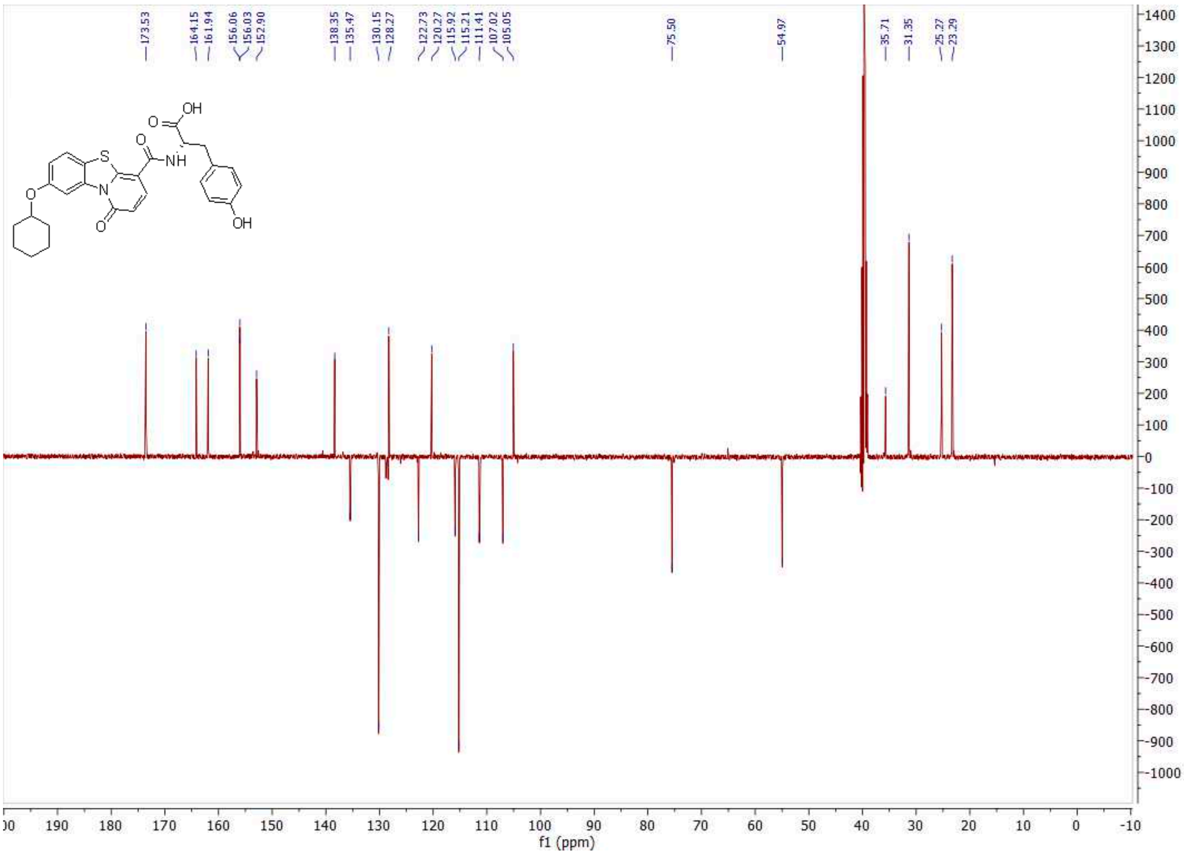

Supplement: Supplementary file 1 [file viruses-13-01585-s001.zip › viruses-1306911-supplementary.pdf]
